# 1,5-Disubstituted Acylated 2-Amino-4,5-dihydroimidazoles as a New Class of Retinoic Acid Receptor–Related Orphan Receptor (ROR) Inhibitors

**DOI:** 10.3390/ijms23084433

**Published:** 2022-04-17

**Authors:** Maria A. Ortiz, F. Javier Piedrafita, Adel Nefzi

**Affiliations:** 1 Donald P. Shiley BioScience Center, San Diego State University, 5500 Campanile Drive, San Diego, CA 92182, USA; maortiz2@sdsu.edu (M.A.O.); fpiedrafita@sdsu.edu (F.J.P.); 2 Center for Translational Science, Florida International University, 11350 SW Village Parkway, Port Saint Lucie, FL 34987, USA

**Keywords:** combinatorial chemistry, parallel synthesis, ROR inhibitors, drug discovery

## Abstract

A growing body of evidence suggests a pathogenic role for pro-inflammatory T helper 17 cells (Th17) in several autoimmune diseases, including multiple sclerosis, rheumatoid arthritis, inflammatory bowel disease, type I diabetes, and psoriasis—diseases for which no curative treatment is currently available. The nuclear retinoic acid receptor–related orphan receptors alpha and gamma (RORα/γ), in particular the truncated isoform RORγt that is specifically expressed in the thymus, play a critical role in the activation of a pro-inflammatory Th17 response, and RORγ inverse agonists have shown promise as negative regulators of Th17 for the treatment of autoimmune diseases. Our study underscores the screening of a large combinatorial library of 1,5-disubstituted acylated 2-amino-4,5-dihydroimidazoles using a demonstrated synthetic and screening approach and the utility of the positional scanning libraries strategy for the rapid identification of a novel class of ROR inhibitors. We identified compound **1295-273** with the highest activity against RORγ (3.3 µM IC_50_) in this series, and almost a two-fold selectivity towards this receptor isoform, with 5.3 and 5.8 µM IC_50_ against RORα and RORβ cells, respectively.

## 1. Introduction

**Figure 1:**
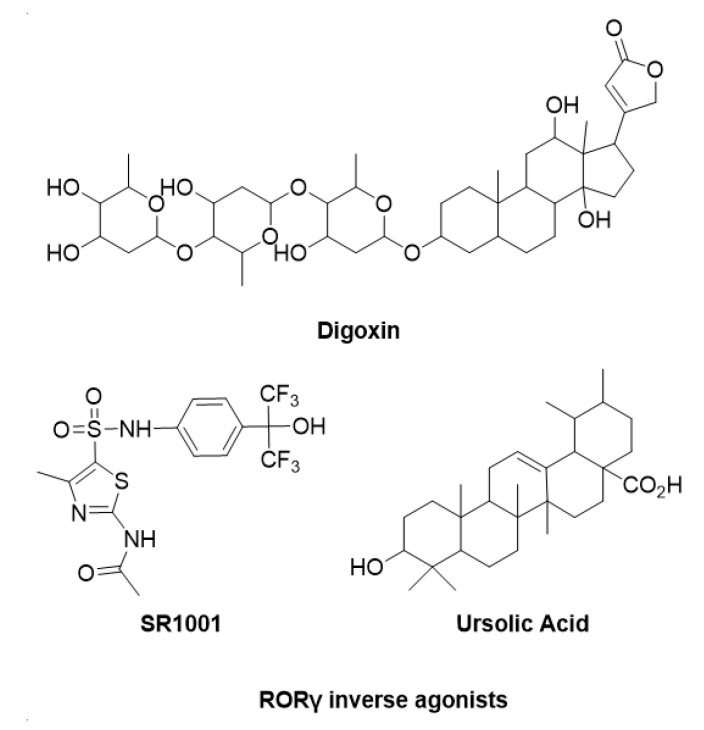
RORγ inverse agonists.

Nuclear hormone receptors are a large family of ligand-dependent transcription factors that play essential roles in regulating many important physiological functions, and they have been implicated in several diseases, including cancer, inflammation, metabolic and autoimmune disorders [1,2]. The retinoic acid receptor–related orphan receptors (RORs), in particular RORα (NR1F1) and RORγt (a truncated form of RORγ (NR1F3) that is specifically expressed in the thymus), are essential for the development of T helper 17 (Th17) cells [3,4]. There is compelling evidence that IL-17-producing Th17 cells play a critical pathogenic role in chronic inflammation and autoimmune diseases, including multiple sclerosis, rheumatoid arthritis, inflammatory bowel disease, type I diabetes, and psoriasis [5,6], which suggested RORs as attractive targets for treating autoimmune disorders. Indeed, it was soon discovered that the RORγ inverse agonists SR1001, digoxin, and ursolic acid could suppress Th17 differentiation and the onset of disease in an experimental autoimmune encephalomyelitis mouse model of multiple sclerosis [7,8,9], Figure 1. 

Crystallographic studies on RORs provided the structural basis for the identification of sterols as ROR ligands [10,11,12]. Besides identifying agonist ligands, most efforts have been dedicated to the identification of ROR antagonists or inverse agonists that could have therapeutic implications. Since then, numerous compounds have been discovered that inhibit RORγ transcriptional activity and are the focus of major therapeutic endeavors by others, including several pharmaceutical companies [13,14]. Here we took advantage of the in-house developed mixture-based chemical libraries to identify novel inhibitors of RORs. The FIU library collection of small molecule compounds consists of more than 30 million compounds designed around 75 molecular scaffolds systematically arranged in positional scanning and scaffold ranking formats [15,16,17,18,19]. Cheminformatic analysis indicates that compared to majority of the commercial libraries, compounds in the FIU libraries are diverse with 3D structures [20,21]. The diversity of the FIU lead generation library has been characterized and described quantitatively by means of molecular scaffolds, molecular properties, and structural fingerprints [17,22]. Fingerprint-based similarity studies demonstrate that individual libraries within the FIU lead generation library occupy highly dense regions in chemical space [23]. The high-density coverage increases the potential of identifying activity cliffs and provides a rapid understanding of the structure–activity relationships (SAR) associated with novel leads and targets [20,21]. Early work from the screening of our compound libraries has shown the utility of mixture-based chemical libraries for the de novo identification of highly active antimicrobial compounds, [18,24] antimalarial, [25,26,27,28,29,30,31] potent analgesic, [15,16,17,18,19,20], antifibrotic compounds, [32] and novel antitumor agents [33,34,35].The FIU library collection thus provides unique lead compounds suitable for optimization for advancement to preclinical and, ultimately, clinical studies, and their successful use have been reviewed [36]. We generated CHO-K1 stable cell lines expressing a 5x-UAS-luciferase reporter and a Gal4-ROR-LBD for all three RORs (α, β, and γ) under the control of a tetracycline repressor. These cell lines were used for the screening of FIU-mixture libraries and we have identified several novel scaffold libraries that could potentially serve as excellent sources for new potent and selective RORγ inhibitors. We identified scaffolds that selectively inhibit binding of RORγ and RORβ but not RORα, such as library **1295**.

This assay is suitable to identify RORγ agonists and antagonists but do not distinguish between the two types of antagonists (classical/neutral antagonists and inverse agonists) [37,38]. RORγ is constitutively activated by endogenous cholesterol-like ligands in the cell, and we used our small-molecule compound library to discover novel RORγ inhibitors that could be suitable for the treatment of autoimmune diseases. 

## 2. Research and Discussion

The 1,5-disubstituted acylated 2-amino-4,5-dihydroimidazole library **1295** is made from 107 mixtures (34 R^1^ × 37 R^2^ × 36 R^3^) and 107 individual control compounds prepared in parallel to the positional scan mixture-based library, [19,25,36,39] and served as controls to verify that the individual building blocks used at each of the variable positions could be successfully incorporated into the synthesis of the mixture libraries. 

**Scheme 1:**
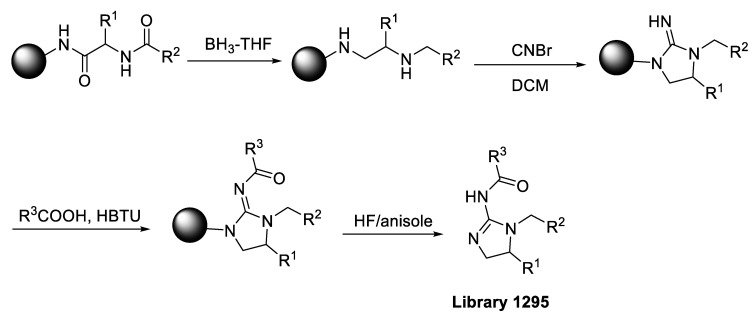
Solid phase parallel synthesis of the 1,5-disubstituted acylated 2-amino-4,5-dihydroimaidazoles.

The R groups making the diversities R^1^, R^2^, and R^3^ were selected in a way that they display a range of different physicochemical properties including hydrophobic, hydrophilic, polar, apolar, basic, acidic, hydrogen acceptors, hydrogen donators, aliphatic and aromatic properties. The synthesis of library **1295** is described in Scheme 1 [40].

**Figure 2:**
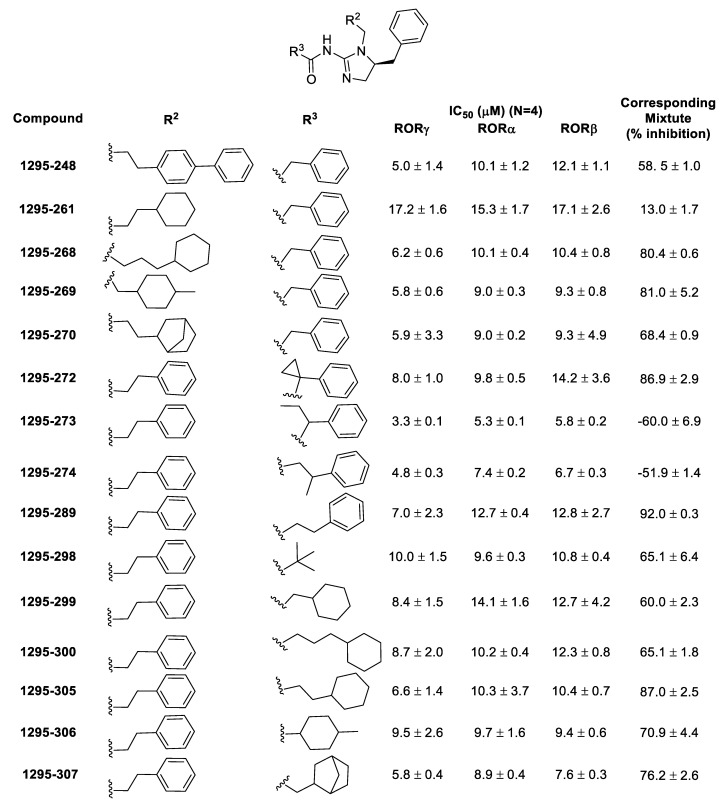
Effect of individual controls from library 1295 on ROR-dependent luciferase activity. The IC50 values (in µM) for each cell line and the chemical group at each position are shown. The right column shows the percentage of inhibition observed in RORγ cells incubated with 10 µg/mL of the corresponding mixture library containing the indicated fixed R2, or R3 functional groups.

We tested the activity of all individual compounds on TRex-CHO-luciferase-RORγ cells at 5 µg/mL (Table S1 in Supplementary Materials); two compounds (**1295-262** and **1295-271**) elicited significant toxicity as determined by a resazurin assay and were discarded from further analysis. Nineteen compounds inhibited doxycycline-dependent luciferase stimulation by more than 80% with no significant toxicity and were titrated in a confirmatory assay with RORγ reporter cells, which afforded IC_50_ values between 1.1 and 4.14 µg/mL. The top 15 compounds from this confirmatory titration experiment, with IC_50_ values below 3 µg/mL, were further evaluated in a second confirmatory dose response experiment with all Gal4-RORγ, RORα, and RORβ reporter cell lines and the IC_50_ (µM) values obtained are shown in Figure 2.

**Figure 3:**
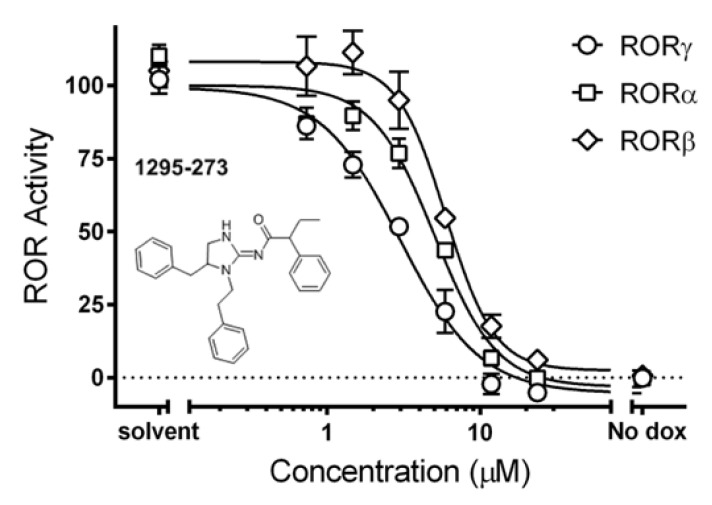
Dose response of compound **1295-273** in ROR cells. TRex-CHO-RORγ/α/β cells were incubated with the indicated concentrations of **1295-273** for 20 h, when cell viability and luciferase activity were determined as indicated in materials and methods. The percentage of normalized activity (luciferase/fluorescence) for each cell line is shown. The experiment was repeated twice with quadruplicate data points and the results of one typical experiment are shown.

Interestingly, all active compounds contain a benzyl group (derived from L-Phe) in position R^1^, which corresponds with one of the most active mixtures with a fixed R^1^. Similarly, a mixture containing phenylacetic acid at position R^2^ was among the most active mixtures with fixed R^2^, which may explain that many active individual compounds also contain such group at position R^2^. In contrast, the mixture with phenylacetic acid at position R^3^ elicited partial activity, which is in agreement with the fact that only a few individual compounds with phenylacetic acid at R^3^ showed activity in RORγ cells. A 2-phenylbutyric acid at position R^3^ bestowed compound **1295-273** with the highest activity against RORγ (3.0 µM IC_50_) in this series, and almost a two-fold selectivity towards this receptor, with 5.04 and 5.94 µM IC_50_ against RORα and RORβ cells, respectively (Figure 3). A 3-phenylbutyric acid at this R^3^ position (compound **1295-274**) was also very active against RORγ cells with an IC_50_ of 4.8 µM and slightly more selective than RORα/β (see Figure 2). This result conflicts with the lack of activity of the corresponding mixture libraries containing 2- or 3-phenylbutyric acid as fixed groups at position R^3^, which were not active when tested at 10 µg/mL (not shown). Another compound worth mentioning is **1295-248**, which showed an IC_50_ of 5 µM and a two-fold selectivity against RORγ cells, with IC_50_ of 10.1 and 12.1 µM against RORα and RORβ cells, respectively. The corresponding mixture with fixed 4-biphenylacetic acid at position R^2^ elicited partial activity (58% inhibition) against RORγ cells.

**Figure 4:**
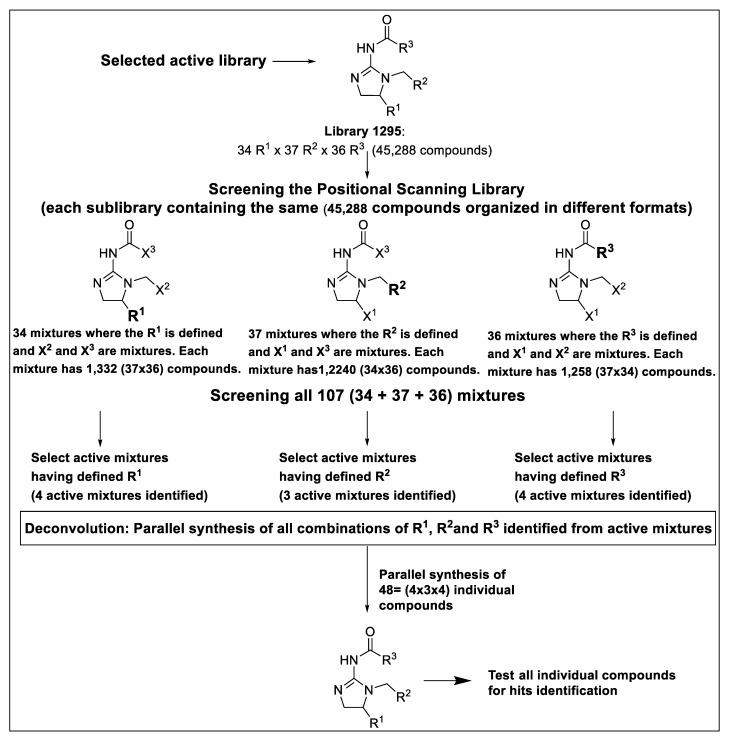
Deconvolution strategy of positional scanning library **1295** for hit identification.

Following the screening at 10 µg/mL and deconvolution of the library **1295** (Figure 4), we prepared 48 compounds (named library **2520**) based on the active mixtures and screened with RORγ cells. Mixtures that showed higher than 75 inhibition and no toxicity in the positional scan were selected for the deconvolution (Table S2 in Supplementary Materials).

**Figure 5:**
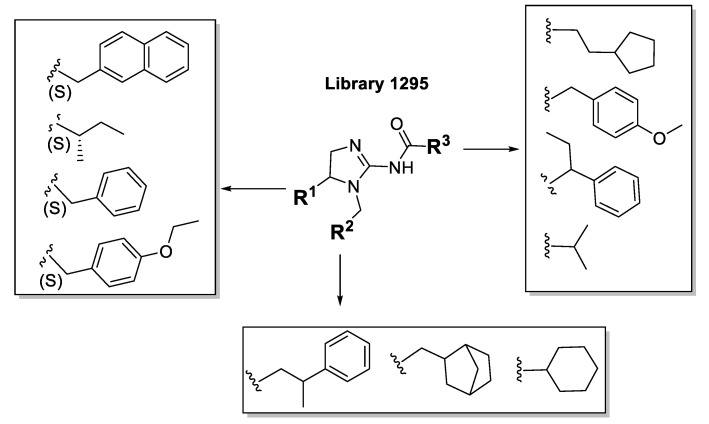
Selected combinations of active mixtures with defined R^1^, R^2^, and R^3^.

The deconvolution of the library **1295** led to four active mixtures with defined position R^1^, three active mixtures with defined position R^2^ and four active mixtures with defined R^3^ with good inhibition activity. The parallel synthesis of all 48 (4 × 3 × 4) individual compounds derived from the deconvolution of library **1295** (Figure 5), representing all the combinations of active mixtures with defined R^1^, R^2^, and R^3^ was performed using the strategy outlined in Scheme 1. 

**Figure 6:**
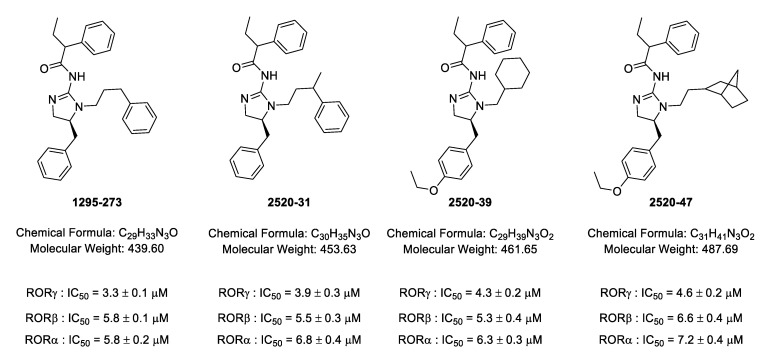
Identified active compounds from the deconvolution of library **1295**. The IC*_50_* of the most active compounds with all three RORs (N = 4) are shown.

The screening data of the synthesized compounds (Table S3 in Supplementary Materials) shows significant differentiation in activity levels among the samples tested at each position, a key feature for deconvoluting a positional scanning library. The deconvolution of the mixtures resulted in compounds with similar potency to compound **1295-273** from the original series of individual controls, but no compounds were identified with improved potency and/or selectivity towards RORγ. Moreover, some preliminary SAR can be seen from the library screening. Aromatic substituents at position R^1^ and R^3^ are important for the activity with the sec-butylbenzene is being preferred at position R^3^. A confirmatory experiment in all three ROR cell lines yielded the following IC_50_ values (Figure 6). 

## 3. Materials and Methods

### 3.1. Chemistry Synthesis

All the reagents, amino acids, and solvents were commercial grade. LC–MS (ESI) traces were recorded on samples with concentrations of 1 mg/ mL in 50:50 MeCN/water at both 214 nm and 254 nm using a reverse phase Vydac column with a gradient of 5 to 95% formic acid in MeCN. The purity of the crude samples was estimated based on the UV traces recorded. Hydrofluoric acid cleaves were performed in specially equipped and ventilated hoods with full personal protective equipment. All synthesized compounds were purified by RP-HPLC (Shimadzu Prominence HPLC system, Kyoto, Japan). The purity of all final compounds was >95%.

The identity and purity of the compounds were verified by Shimatzu HPLC and mass spectra under the following conditions; column, Phenomenex Luna 150 × 21.20 mm, 5 micron, C18; mobile phase, (A) H_2_O (+0.1% formic acid)/(B) MeCN (+0.1% formic acid), and 3 gradient methods used based on compound hydrophobicity (2% B to 20% B, 11 min; 25% B to 45% B, 31 min; 45% B to 65% B, 21 min); flow rate, 12 mL/min; detection, UV 214 nm. All chirality data were generated from the corresponding amino acids. Under our reaction conditions the epimerization was minimized to less than 5% [28,41,42,43].

General Synthesis of the 1,5-disubstituted acylated 2-amino-4,5-dihydroimidazole compounds: All individual libary-**1295** and individual library-**2520** compounds were synthesized following the strategy outlined in Scheme 1 [40]. The solid phase synthesis was performed using the “tea-bag” methodology [44,45]. 100 mg of p-methylbenzdrylamine (pMBHA) resin per compound (1.1 mmol/g, 100–200 mesh) was sealed in a mesh “tea-bag”, neutralized with 5% diisopropylethylamine (DIEA) in dichloromethane (DCM), and subsequently swelled with additional DCM washes. The first diversity R^1^ was introduced by the coupling of Boc amino acid (6 equiv.) in dimethylformamide (0.1M DMF) for 60 min in the presence of diisopropylcarbodiimide (DIC, 6 equiv.) and 1-hydroxybenzotriazole hydrate (HOBt, 6 equiv.). The Boc-protecting group was removed with 55% TFA/DCM for 30 minutes and subsequently neutralized with 5% DIEA/DCM (3×). The second diversity (R^2^) was introduced by the subsequent coupling of a carboxylic (10 equiv.) in the presence of DIC (10 equiv.) overnight in DMF anhydrous. All coupling reactions were monitored for completion by Ninhydrin test [46].

The reduction of the amide bonds was performed in a 1000 mL Wilmad LabGlass vessel under nitrogen in the presence of 1.0M borane–tetrahydrofuran (BH_3_-THF) complex solution (40-fold excess of BH_3_-THF per amide bond). The reaction vessel was heated to 65 °C and the temperature was maintained for 72 h [42,47]. The solution was then discarded, and the bags were washed with THF and methanol. Once completely dry, the bags were treated overnight with piperidine at 65 °C and washed several times with methanol, DMF and DCM. Before proceeding, completion of the reduction was monitored by a control cleavage and analyzed by LC–MS.

The generated resin bound diamines were treated with cyanogen bromide (CNBr) (5 eq) in anhydrous dichloromethane (DCM) overnight. The generated cyclic guanidines were treated with a carboxylic acid (10 eq) in the presence of chloroacetone (10 eq) in DMSO in the presence of 3-[Bis(dimethylamino)methyliumyl]-3H-benzotriazol-1-oxide hexafluorophosphate (HBTU) (10 eq) and DIEA (10 eq) overnight. The desired 1,5-disubstituted acylated 2-amino-4,5-dihydroimaidazole compounds were cleaved from the solid-support in the presence of HF and then extracted with acetic acid and lyophilized to obtain solid white powder. The positional scanning library was synthesized using the same strategy.

### 3.2. Cell Lines and Luciferase Assays

To investigate the transcriptional activity of all three RORs, we generated stable cell lines expressing an UAS-driven luciferase reporter together with the corresponding Gal4-DNA binding domain fused to the ligand binding domains (LBDs) of mRORγ (Pro-261-end), hRORα (Pro-211-end), or rRORβ (Pro 205-end). The Gal4-DBD-ROR-LBD fusion proteins were subcloned in the pcDNA4/TO expression vector (Thermo Scientific) and transfected into TRex-CHO-K1 cells (Thermo Scientific) for tetracycline-controlled expression of the corresponding ROR proteins. Transfected cells were selected with hygromycin and Zeocin and individual clones for each of the Gal4-RORγ, Gal4-RORα, and Gal4-RORβ cell lines were isolated using a standard serial dilution protocol. Clones with the highest doxycycline-dependent stimulation of luciferase activity were selected for all functional experiments. All cell lines were maintained in complete Ham’s F12 medium containing 10% fetal bovine serum (FBS), 10 µg/mL blasticidine, 100 µg/mL hygromycin, and 100 µg/mL Zeocin and were used within 2 months of culture without any loss of doxycycline-dependent stimulation of luciferase activity.

For screening FIU libraries, cells were resuspended in Ham’s F-12 assay medium containing 2% charcoal-treated FBS and no selection drugs. Ten thousand cells per well were seeded in 30 µL of assay medium in 384 white plates with clear bottom (Greiner). Cells were allowed to attach for 2 h, followed by compound treatment and stimulation with 20 ng/mL doxycycline for an additional 16–20 h. Appropriate solvent controls, negative controls with no doxycycline, and blanks with no cells were included in every plate. Prior to measuring luciferase activity, 6 µL of 0.15 mg/mL resazurin (Cayman Chemical) in PBS were added and cells were incubated for 1 h at 37 °C. Fluorescence intensity was detected at 545 nm excitation/590 nm emission using a CLARIOstar reader (BMGLabtech) as a measure of cell viability, to identify any potential cytotoxic effect of test compounds and mixtures. Following the resazurin assay, the medium was removed and 20 µL of BriteLite-Plus luciferase reagent (Perking Elmer) were added; luminescence was measured in a CLARIOstar reader using a 384 aperture spoon and a 0.1 sec measuring time interval.

Luminescence was normalized by the fluorescence intensity and the percentage of inhibition was calculated with respect to control cells stimulated with doxycycline in the presence of solvent control (0.1% DMF). All primary screens with individual compounds were performed in triplicate with a final concentration of 5 µg/mL. Hits with more than 50% inhibition were subjected to a dose-response confirmatory assay followed by a second 6-point titration experiment with quadruplicate data points. IC_50_ values were calculated using GraphPrism (GraphPad software).

## 4. Conclusions 

In conclusion, the screening of a large complex library of 1,5-Disubstituted acylated 2-amino-4,5-dihydroimidazoles using a demonstrated synthetic and screening approach led to the rapid identification of novel structurally unique inhibitors of the nuclear retinoic acid receptor–related orphan receptors (RORs) with slight selectivity towards RORγ. We found that aromatic substituents are favored at positions R^1^ and R^3^ while alkyl, aryl, and cycloalkyl substituents are preferred in position R^2^. Our study underscores the utility of the positional scanning libraries in rapid identification of novel ROR inhibitors. This class of compounds have the promise to be effective for controlling inflammatory autoimmunity therapy. Future studies will focus on structure-based computational design efforts to further improve the potency and the RORγ selectivity of the identified hits.

## Data Availability

Not applicable.

## Supplementary Materials

The following supporting information can be downloaded at: www.mdpi.com/article/10.3390/ijms23084433/s1.

## Abbreviation

ESI–MS: electrospray ionization mass spectrometry; LC–MS: liquid chromatography coupled to mass spectrometry; RP-HPLC: reverse phase high-performance liquid chromatography; TFA: trifluoroacetic acid; UV: ultraviolet; DCM: dichloromethane; DMF: dimethyl formamide; THF: tetrahydrofuran; HF: hydrogen anhydrous.

## References

[1] Mangelsdorf D.J., Thummel C., Beato M., Herrlich P., Schütz G., Umesono K., Blumberg B., Kastner P., Mark M., Chambon P. (1995). The nuclear receptor superfamily: The second decade. Cell.

[2] Burris T.P., Busby S.A., Griffin P.R. (2012). Targeting orphan nuclear receptors for treatment of metabolic diseases and autoimmunity. Chem. Biol..

[3] Ivanov I.I., McKenzie B.S., Zhou L., Tadokoro C.E., Lepelley A., Lafaille J.J., Cua D.J., Littman D.R. (2006). The orphan nuclear receptor RORgammat directs the differentiation program of proinflammatory IL-17+ T helper cells. Cell.

[4] Yang X.O., Pappu B.P., Nurieva R., Akimzhanov A., Kang H.S., Chung Y., Ma L., Shah B., Panopoulos A.D., Schluns K.S. (2008). T helper 17 lineage differentiation is programmed by orphan nuclear receptors ROR alpha and ROR gamma. Immunity.

[5] Waite J.C., Skokos D. (2012). Th17 response and inflammatory autoimmune diseases. Int. J. Inflam..

[6] Miossec P., Kolls J.K. (2012). Targeting IL-17 and TH17 cells in chronic inflammation. Nat. Rev. Drug Discov..

[7] Huh J.R., Leung M.W., Huang P., Ryan D.A., Krout M.R., Malapaka R.R., Chow J., Manel N., Ciofani M., Kim S.V. (2011). Digoxin and its derivatives suppress TH17 cell differentiation by antagonizing RORγt activity. Nature.

[8] Solt L.A., Kumar N., Nuhant P., Wang Y., Lauer J.L., Liu J., Istrate M.A., Kamenecka T.M., Roush W.R., Vidović D. (2011). Suppression of TH17 differentiation and autoimmunity by a synthetic ROR ligand. Nature.

[9] Xu T., Wang X., Zhong B., Nurieva R.I., Ding S., Dong C. (2011). Ursolic acid suppresses interleukin-17 (IL-17) production by selectively antagonizing the function of RORgamma t protein. J. Biol. Chem..

[10] Kallen J.A., Schlaeppi J.M., Bitsch F., Geisse S., Geiser M., Delhon I., Fournier B. (2002). X-ray structure of the hRORalpha LBD at 1.63 A: Structural and functional data that cholesterol or a cholesterol derivative is the natural ligand of RORalpha. Structure.

[11] Kallen J., Schlaeppi J.M., Bitsch F., Delhon I., Fournier B. (2004). Crystal structure of the human RORalpha Ligand binding domain in complex with cholesterol sulfate at 2.2 A. J. Biol. Chem..

[12] Jin L., Martynowski D., Zheng S., Wada T., Xie W., Li Y. (2010). Structural basis for hydroxycholesterols as natural ligands of orphan nuclear receptor RORgamma. Mol. Endocrinol..

[13] Fauber B.P., Magnuson S. (2014). Modulators of the nuclear receptor retinoic acid receptor-related orphan receptor-γ (RORγ or RORc). J. Med. Chem..

[14] Huang M., Bolin S., Miller H., Ng H.L. (2020). RORγ Structural Plasticity and Druggability. Int. J. Mol. Sci..

[15] Wu J., Zhang Y., Maida L.E., Santos R.G., Welmaker G.S., LaVoi T.M., Nefzi A., Yu Y., Houghten R.A., Toll L. (2013). Scaffold ranking and positional scanning utilized in the discovery of nAChR-selective compounds suitable for optimization studies. J. Med. Chem..

[16] Al-Ali H., Debevec G., Santos R.G., Houghten R.A., Davis J.C., Nefzi A., Lemmon V.P., Bixby J.L., Giulianotti M.A. (2018). Scaffold Ranking and Positional Scanning Identify Novel Neurite Outgrowth Promoters with Nanomolar Potency. ACS Med. Chem. Lett..

[17] Singh N., Guha R., Giulianotti M.A., Pinilla C., Houghten R.A., Medina-Franco J.L. (2009). Chemoinformatic analysis of combinatorial libraries, drugs, natural products, and molecular libraries small molecule repository. J. Chem. Inf. Model..

[18] Fleeman R., LaVoi T.M., Santos R.G., Morales A., Nefzi A., Welmaker G.S., Medina-Franco J.L., Giulianotti M.A., Houghten R.A., Shaw L.N. (2015). Combinatorial Libraries As a Tool for the Discovery of Novel, Broad-Spectrum Antibacterial Agents Targeting the ESKAPE Pathogens. J. Med. Chem..

[19] Chesnokov O., Visitdesotrakul P., Kalani K., Nefzi A., Oleinikov A.V. (2021). Small Molecule Compounds Identified from Mixture-Based Library Inhibit Binding between Plasmodium falciparum Infected Erythrocytes and Endothelial Receptor ICAM-1. Int. J. Mol. Sci..

[20] López-Vallejo F., Nefzi A., Bender A., Owen J.R., Nabney I.T., Houghten R.A., Medina-Franco J.L. (2011). Increased diversity of libraries from libraries: Chemoinformatic analysis of bis-diazacyclic libraries. Chem. Biol. Drug Des..

[21] López-Vallejo F., Caulfield T., Martínez-Mayorga K., Giulianotti M.A., Nefzi A., Houghten R.A., Medina-Franco J.L. (2011). Integrating virtual screening and combinatorial chemistry for accelerated drug discovery. Comb. Chem. High Throughput Screen.

[22] Jose L.M.-F., Karina M.-M., Marc A.G., Richard A.H., Clemencia P. (2008). Visualization of the Chemical Space in Drug Discovery. Curr. Comput. Aided Drug Des..

[23] Medina-Franco J.L., Martínez-Mayorga K., Bender A., Marín R.M., Giulianotti M.A., Pinilla C., Houghten R.A. (2009). Characterization of Activity Landscapes Using 2D and 3D Similarity Methods: Consensus Activity Cliffs. J. Chem. Inf. Model..

[24] Hensler M.E., Bernstein G., Nizet V., Nefzi A. (2006). Pyrrolidine bis-cyclic guanidines with antimicrobial activity against drug-resistant Gram-positive pathogens identified from a mixture-based combinatorial library. Bioorg. Med. Chem. Lett..

[25] Perry D.L., Roberts B.F., Debevec G., Michaels H.A., Chakrabarti D., Nefzi A. (2019). Identification of Bis-Cyclic Guanidines as Antiplasmodial Compounds from Positional Scanning Mixture-Based Libraries. Molecules.

[26] Dellai A., Appel J., Bouraoui A., Croft S., Nefzi A. (2013). Antimalarial and cytotoxic activities of chiral triamines. Bioorg. Med. Chem. Lett..

[27] Houghten R.A., Ganno M.L., McLaughlin J.P., Dooley C.T., Eans S.O., Santos R.G., LaVoi T., Nefzi A., Welmaker G., Giulianotti M.A. (2016). Direct Phenotypic Screening in Mice: Identification of Individual, Novel Antinociceptive Compounds from a Library of 734,821 Pyrrolidine Bis-piperazines. ACS Comb. Sci..

[28] Nefzi A., Ostresh J.M., Appel J.R., Bidlack J., Dooley C.T., Houghten R.A. (2006). Identification of potent and highly selective chiral tri-amine and tetra-amine mu opioid receptors ligands: An example of lead optimization using mixture-based libraries. Bioorg. Med. Chem. Lett..

[29] Dooley C.T., Ny P., Bidlack J.M., Houghten R.A. (1998). Selective ligands for the mu, delta, and kappa opioid receptors identified from a single mixture based tetrapeptide positional scanning combinatorial library. J. Biol. Chem..

[30] Yongye A.B., Pinilla C., Medina-Franco J.L., Giulianotti M.A., Dooley C.T., Appel J.R., Nefzi A., Scior T., Houghten R.A., Martínez-Mayorga K. (2011). Integrating computational and mixture-based screening of combinatorial libraries. J. Mol. Model..

[31] Reilley K.J., Giulianotti M., Dooley C.T., Nefzi A., McLaughlin J.P., Houghten R.A. (2010). Identification of two novel, potent, low-liability antinociceptive compounds from the direct in vivo screening of a large mixture-based combinatorial library. AAPS J..

[32] Stefanovic B., Michaels H.A., Nefzi A. (2021). Discovery of a Lead Compound for Specific Inhibition of Type I Collagen Production in Fibrosis. ACS Med. Chem. Lett..

[33] Hastings R.H., Burton D.W., Nefzi A., Montgrain P.R., Quintana R., Deftos L.J. (2010). Combinatorial library discovery of small molecule inhibitors of lung cancer proliferation and parathyroid hormone-related protein expression. Cancer Biol. Ther..

[34] Schimmer A.D., Welsh K., Pinilla C., Wang Z., Krajewska M., Bonneau M.J., Pedersen I.M., Kitada S., Scott F.L., Bailly-Maitre B. (2004). Small-molecule antagonists of apoptosis suppressor XIAP exhibit broad antitumor activity. Cancer Cell.

[35] Carter B.Z., Gronda M., Wang Z., Welsh K., Pinilla C., Andreeff M., Schober W.D., Nefzi A., Pond G.R., Mawji I.A. (2005). Small-molecule XIAP inhibitors derepress downstream effector caspases and induce apoptosis of acute myeloid leukemia cells. Blood.

[36] Houghten R.A., Pinilla C., Appel J.R., Blondelle S.E., Dooley C.T., Eichler J., Nefzi A., Ostresh J.M. (1999). Mixture-based synthetic combinatorial libraries. J. Med. Chem..

[37] Gronemeyer H., Gustafsson J.A., Laudet V. (2004). Principles for modulation of the nuclear receptor superfamily. Nat. Rev. Drug Discov..

[38] Bourguet W., de Lera A.R., Gronemeyer H. (2010). Inverse agonists and antagonists of retinoid receptors. Methods Enzymol..

[39] Pinilla C., Appel J.R., Blanc P., Houghten R.A. (1992). Rapid identification of high affinity peptide ligands using positional scanning synthetic peptide combinatorial libraries. Biotechniques.

[40] Acharya A.N., Ostresh J.M., Houghten R.A. (2001). A novel approach for the solid-phase synthesis of substituted cyclic guanidines, their respective bis analogues, and N-acylated guanidines from N-acylated amino acid amides. J. Comb. Chem..

[41] Nefzi A., Giulianotti M.A., Houghten R.A. (2000). Solid-Phase Synthesis of Substituted 2,3-Diketopiperazines from Reduced Polyamides. Tetrahedron.

[42] Nefzi A., Giulianotti M.A., Ong N.A., Houghten R.A. (2000). Solid-phase synthesis of bis-2-imidazolidinethiones from resin-bound tripeptides. Org. Lett..

[43] Ostresh J.M., Schoner C.C., Hamashin V.T., Nefzi A., Meyer J.-P., Houghten R.A. (1998). Solid-Phase Synthesis of Trisubstituted Bicyclic Guanidines via Cyclization of Reduced N-Acylated Dipeptides. J. Org. Chem..

[44] Houghten R.A. (1985). General method for the rapid solid-phase synthesis of large numbers of peptides: Specificity of antigen-antibody interaction at the level of individual amino acids. Proc. Natl. Acad. Sci. USA.

[45] Nefzi A., Ostresh J.M., Yu Y., Houghten R.A. (2004). Combinatorial chemistry: Libraries from libraries, the art of the diversity-oriented transformation of resin-bound peptides and chiral polyamides to low molecular weight acyclic and heterocyclic compounds. J. Org. Chem..

[46] Kaiser E., Colescott R.L., Bossinger C.D., Cook P.I. (1970). Color test for detection of free terminal amino groups in the solid-phase synthesis of peptides. Anal. Biochem..

[47] Nefzi A., Giulianotti M.A., Houghten R.A. (2001). Solid-phase synthesis of bis-heterocyclic compounds from resin-bound orthogonally protected lysine. J. Comb. Chem..

