# Supplementary material for: 1,5-Disubstituted Acylated 2-Amino-4,5-dihydroimidazoles as a New Class of Retinoic Acid Receptor–Related Orphan Receptor (ROR) Inhibitors
Source: Int J Mol Sci. 2022 Apr 17;23(8):4433. doi: 10.3390/ijms23084433 (PMC9029089; doi:10.3390/ijms23084433)
Supplement: Supplementary file 1 [file ijms-23-04433-s001.zip › ijms-1662185-supplementary.pdf]

1,5-Disubstituted acylated 2-amino-4,5-dihydroimidazoles as a new class of retinoic acid receptor-related orphan receptor (ROR) inhibitors

Maria A. Ortiz [1], F. Javier Piedrafita [1], and Adel Nefzi [2 *]

[1] Donald P. Shiley BioScience Center, San Diego State University, 5500 Campanile Drive, San Diego, California 92182-4650

[2] Center for Translational Science, Florida International University, 11350 SW Village Parkway, Port Saint Lucie, FL 34987

Supplementary material

Distribution list and activity of the positional scanning library **1295.**

Distribution list and activity of the individual controls **1295.**

Distribution list and activity of the compounds 2520 derived from the deconvolution of the mixture-based library.

LCMS of the selected active compounds

Table S1: distribution list of library **1295**

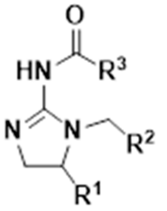

**Library 1295**	Acylated cyclic guanidine PS-SCL		**Total diversity: 45,288 compounds**			
	**R1**	**R2**	**R3**	**Nomenclature**	**% Inhibition**	**IC_{50}**
Functional group	34 amino acids	37 carboxylic acids	36 carboxylic acids		at 10 µg/ml	in µg/ml
Compounds per mixture	1,332 (37 x 36)	1,224 (34 x 36)	1,258 (37 x 34)		N=3	N=3
1295.001	Boc-L-Ala	X	X	S-methyl	59.4 ± 1.3	
1295.002	Boc-L-Phe	X	X	S-benzyl	94.1 ± 0.5	2.054 ± 0.166
1295.003	Boc-Gly	X	X	hydrogen	35.0 ± 4.7	
1295.004	Boc-L-Ile	X	X	S-2-butyl	22.6 ± 2.8	
1295.005	Boc-L-Leu	X	X	S-isobutyl	-23.7 ± 1.4	
1295.006	Boc-L-Val	X	X	S-isopropyl	13.4 ± 2.4	
1295.007	Boc-L-Tyr(BrZ)	X	X	S-4-hydroxybenzyl	4.3 ± 2.9	
1295.008	Boc-D-Ala	X	X	R-methyl	48.6 ± 2.7	
1295.009	Boc-D-Phe	X	X	R-benzyl	98.7 ± 2.3	2.257 ± 0.247
1295.010	Boc-D-Ile	X	X	R-2-butyl	88.6 ± 2.3	2.024 ± 0.181
1295.011	Boc-D-Leu	X	X	R-isobutyl	97.6 ± 1.8	1.684 ± 0.114
1295.012	Boc-D-Val	X	X	R-isopropyl	77.5 ± 3.1	
1295.013	Boc-D-Tyr(BrZ)	X	X	S-4-hydroxybenzyl	55.9 ± 7.0	
1295.014	Boc-a-aminoisobutyric acid	X	X	2 different methyls at R1	64.6 ± 7.8	
1295.015	Boc-L-Norvaline	X	X	S-propyl	72.4 ± 7.8	
1295.016	Boc-D-Norvaline	X	X	R-propyl	84.7 ± 3.8	3.118 ± 0.476
1295.017	Boc-L-Norleucine	X	X	S-butyl	70.6 ± 6.5	
1295.018	Boc-D-Norleucine	X	X	R-butyl	39.4 ± 4.7	
1295.019	Boc-L-Phenylglycine	X	X	S-phenyl	12.7 ± 8.4	
1295.020	Boc-D-Phenylglycine	X	X	R-phenyl	69.5 ± 4.2	
1295.021	Boc-L-Naphthylalanine	X	X	S-2-naphthylmethyl	26.7 ± 4.4	
1295.022	Boc-D-Naphthylalanine	X	X	R-2-naphthylmethyl	57.3 ± 11.5	
1295.023	Boc-L-cyclohexylAlanine	X	X	S-cyclohexyl	91.3 ± 3.1	1.801 ± 0.157
1295.024	Boc-D-cyclohexylAlanine	X	X	R-cyclohexyl	84.8 ± 1.8	2.672 ± 0.324
1295.025	Boc-L-4-Chlorophenylalanine	X	X	S-4-chloro-benzyl	86.3 ± 0.3	2.294 ± 0.262
1295.026	Boc-D-4-Chlorophenylalanine	X	X	R-4-chloro-benzyl	46.6 ± 0.0	

1295.027	Boc-L-4-Fluorophenylalanine	X	X	S-4-fluoro-benzyl	39.9 ± 9.5	
1295.028	Boc-D-4-Fluorophenylalanine	X	X	R-4-fluoro-benzyl	90.7 ± 0.9	1.923 ± 0.131
1295.029	Boc-L-(3-pyridyl)alanine	X	X	S-pyridin-3-yl-methyl	-3.2 ± 7.4	
1295.030	Boc-D-(3-pyridyl)alanine	X	X	R-pyridin-3-yl-methyl	21.1 ± 12.0	
1295.031	Boc-L-a-tButylglycine	X	X	S-tert-butyl	87.4 ± 1.9	3.284 ± 0.254
1295.032	Boc-D-a-tbutylglycine	X	X	R-tert-butyl	93.4 ± 3.2	2.914 ± 0.322
1295.033	Boc-O-ethyl-L-Tyrosine	X	X	S-4-ethoxy-benzyl	94.2 ± 0.8	1.916 ± 0.179
1295.034	Boc-O-ethyl-D-Tyrosine	X	X	R-4-ethoxy-benzyl	91.8 ± 0.1	2.018 ± 0.151
1295.035	X	2-phenylbutyric acid	X	2-phenylbutyl	67.8 ± 2.8	
1295.036	X	3-Phenylbutyric Acid	X	3-phenylbutyl	66.1 ± 1.6	
1295.037	X	m-Tolylacetic Acid	X	*m*-tolylethyl	75.9 ± 1.2	2.615 ± 0.230
1295.038	X	3-Fluorophenylacetic Acid	X	2-(3-fluoro-phenyl)-ethyl	5.4 ± 3.8	
1295.039	X	3-Bromophenylacetic Acid	X	2-(3-bromo-phenyl)-ethyl	55.8 ± 3.1	
1295.040	X	4-Fluorophenylacetic Acid	X	2-(4-fluoro-phenyl)-ethyl	60.9 ± 0.6	
1295.041	X	3-Methoxyphenylacetic Acid	X	2-(3-methoxy-phenyl)-ethyl	-7.4 ± 0.4	
1295.042	X	4-Bromophenylacetic Acid	X	2-(4-bromo-phenyl)-ethyl	49.1 ± 1.3	
1295.043	X	4-Methoxyphenylacetic Acid	X	2-(4-methoxy-phenyl)-ethyl	-1.4 ± 2.2	
1295.044	X	4-Ethoxyphenylacetic Acid	X	2-(4-ethoxy-phenyl)-ethyl	48.7 ± 3.7	
1295.045	X	4-Isobutyl-alpha-Methylphenylacetic Acid	X	2-(4-Isobutyl-phenyl)-propyl	67.3 ± 1.0	
1295.046	X	3,5-Bis(Trifluoromethyl)-Phenylacetic Acid	X	2-(3,5-bis-trifluoromethyl-phenyl)-ethyl	63.0 ± 1.1	
1295.047	X	3-(3,4-Dimethoxyphenyl)-Propionic Acid	X	3-(3,4-dimethoxy-phenyl)-propyl	81.2 ± 0.2	1.246 ± 0.252
1295.048	X	4-Biphenylacetic Acid	X	2-Biphenyl-4-yl-ethyl	58.5 ± 1.0	
1295.049	X	(3,4-Dimethoxyphenyl) Acetic Acid	X	2-(3,4-dimethoxyphenyl)-ethyl	14.1 ± 2.8	
1295.050	X	Phenylacetic Acid	X	phenethyl	78.9 ± 1.3	1.771 ± 0.132
1295.051	X	Hydrocinnamic Acid	X	phenylpropyl	80.8 ± 1.1	2.123 ± 0.142

1295.052	X	4-Phenylbutyric Acid	X	4-phenylbutyl	25.7	± 4.3	
1295.053	X	Butyric Acid	X	butyl	48.0	± 0.6	
1295.054	X	Heptanoic Acid	X	heptyl	-4.2	± 2.5	
1295.055	X	Isobutyric Acid	X	isobutyl	-28.1	± 0.5	
1295.056	X	(+/-)-2-Methylbutyric Acid	X	(+/-)-2-Methylbutyl	-4.1	± 13.0	
1295.057	X	Isovaleric Acid	X	3-methylbutyl	24.6	± 5.3	
1295.058	X	4-Methylvaleric Acid	X	4-methylpentyl	-8.6	± 6.8	
1295.059	X	Trimethylacetic Acid	X	*t*-butylmethyl	10.4	± 9.6	
1295.060	X	Cyclohexanecarboxylic Acid	X	cyclohexyl-methyl	87.5	± 0.3	2.507 ± 0.245
1295.061	X	Cyclohexylacetic Acid	X	cyclohexyl-ethyl	13.0	± 1.7	
1295.062	X	Cyclohexanebutyric Acid	X	cyclohexyl-butyl	10.4	± 2.3	
1295.063	X	Cycloheptanecarboxylic Acid	X	cycloheptyl-methyl	44.0	± 9.5	
1295.064	X	Acetic Acid	X	ethyl	-47.8	± 11.8	
1295.065	X	Cyclobutanecarboxylic Acid	X	cyclobutyl-methyl	-62.4	± 14.0	
1295.066	X	Cyclopentanecarboxylic Acid	X	cyclopentyl-methyl	-67.2	± 7.5	
1295.067	X	3-Cyclopentylpropionic Acid	X	3-cyclopentyl-propyl	-2.1	± 36.7	
1295.068	X	Cyclohexanepropionic Acid	X	cyclohexyl-propyl	80.4	± 0.6	2.582 ± 0.201
1295.069	X	4-Methyl-1-Cyclohexanecarboxylic Acid	X	4-methyl-1-cyclohexylmethyl	81.0	± 5.2	2.038 ± 0.126
1295.070	X	2-Norbornaneacetic Acid	X	2-Bicyclo[2.2.1]hept-2-yl-ethyl	68.4	± 0.9	
1295.071	X	1-Adamantaneacetic Acid	X	2-adamantan-1-yl-ethyl	74.7	± 1.4	2.034 ± 0.125
1295.072	X	X	1-phenyl-1cyclopropanecarboxylic acid	1-phenyl-cyclopropyl	86.9	± 2.9	1.957 ± 0.181
1295.073	X	X	2-Phenylbutyric Acid	1-phenyl-propyl	-60.0	± 6.9	
1295.074	X	X	3-Phenylbutyric Acid	2-phenyl-propyl	-51.9	± 14.0	
1295.075	X	X	m-Tolylacetic Acid	4-methyl-benzyl	65.1	± 6.0	
1295.076	X	X	3-Fluorophenylacetic Acid	3-fluoro-benzyl	53.7	± 13.5	
1295.077	X	X	3-Bromophenylacetic Acid	3-bromo-benzyl	50.4	± 2.2	
1295.078	X	X	4-Fluorophenylacetic Acid	4-fluoro-benzyl	68.4	± 8.5	
1295.079	X	X	3-Methoxyphenylacetic Acid	3-methoxy-benzyl	26.2	± 6.3	
1295.080	X	X	4-Bromophenylacetic Acid	4-bromo-benzyl	-4.5	± 21.2	

1295.081	X	X	4-Methoxyphenylacetic Acid	4-methoxy-benzyl	50.5 ± 2.7	
1295.082	X	X	4-Ethoxyphenylacetic Acid	4-ethoxy-benzyl	54.8 ± 3.8	
1295.083	X	X	4-Isobutyl-alpha-Methylphenylacetic Acid	4-isobutyl-benzyl	95.2 ± 0.1	1.783 ± 0.124
1295.084	X	X	3-(3,4-Dimethoxyphenyl)-Propionic Acid	2-(3,4-dimethoxy-phenyl)-ethyl	87.5 ± 0.9	3.769 ± 0.420
1295.085	X	X	4-Biphenylacetic Acid	Biphenyl-4-yl-methyl	73.7 ± 0.6	
1295.086	X	X	2-(Trifluoromethyl)-cinnamic Acid	2-(2-trifluoromethyl-phenyl)-vinyl	95.6 ± 0.5	2.062 ± 0.151
1295.087	X	X	(3,4-Dimethoxyphenyl) Acetic Acid	3,4-dimethoxy-benzyl	75.6 ± 2.7	
1295.088	X	X	m-toluic acid	3-methyl-phenyl	47.3 ± 0.0	
1295.089	X	X	Hydrocinnamic Acid	2-phenyl-ethyl	92.0 ± 0.3	3.458 ± 0.283
1295.090	X	X	4-Phenylbutyric Acid	3-phenyl-propyl	72.1 ± 3.7	
1295.091	X	X	phenylacetic acid	benzyl	52.8 ± 1.5	
1295.092	X	X	4-Ethyl-4-Biphenylcarboxylic Acid	4'-ethyl-biphenyl-4-yl	-7.7 ± 0.4	
1295.093	X	X	Butyric Acid	propyl	32.9 ± 1.6	
1295.094	X	X	Heptanoic Acid	hexyl	89.7 ± 2.8	3.413 ± 0.191
1295.095	X	X	Isobutyric Acid	isopropyl	64.7 ± 2.7	
1295.096	X	X	(+/-)-2-Methylbutyric Acid	1-methyl-propyl	51.0 ± 3.4	
1295.097	X	X	4-Methylvaleric Acid	3-methyl-butyl	75.3 ± 6.8	
1295.098	X	X	Trimethylacetic Acid	*tert*-butyl	65.1 ± 6.4	
1295.099	X	X	Cyclohexylacetic Acid	cyclohexyl-methyl	60.0 ± 2.3	
1295.100	X	X	Cyclohexanebutyric Acid	cyclohexyl-propyl	65.1 ± 1.8	
1295.101	X	X	Acetic Acid	ethyl	38.2 ± 3.2	
1295.102	X	X	Cyclobutanecarboxylic Acid	cyclobutyl	0.2 ± 8.8	
1295.103	X	X	Cyclopentanecarboxylic Acid	cyclopentyl	22.0 ± 16.6	
1295.104	X	X	3-Cyclopentylpropionic Acid	2-cyclopentyl-ethyl	76.0 ± 4.3	
1295.105	X	X	Cyclohexanepropionic Acid	2-cyclohexyl-ethyl	87.0 ± 2.5	2.721 ± 0.164
1295.106	X	X	4-Methyl-1-Cyclohexanecarboxylic Acid	4-methyl-cyclohexyl	70.9 ± 4.4	
1295.107	X	X	2-Norbornaneacetic Acid	Bicyclo[2.2.1]hept-2-yl-methyl	76.2 ± 2.6	3.281 ± 0.322

X = equimolar mixture of aminoacids (R1) or carboxylic acids (R2 and R3)

Table S2: Individual Controls prepared in parallel to the synthesis of library 1295

TPI 1295 Acylated cyclic guanidine

Controls: 201-307

bag no.	R1	R2	R3	mol wt.	% Inhibition RORγ 5 μg/ml
1295.201	Boc-L-Ala	Phenylacetic Acid	phenylacetic acid	321.2	-40.7 ± 14.9
1295.202	Boc-L-Phe	Phenylacetic Acid	phenylacetic acid	397.2	0.0 ± 7.2
1295.203	Boc-Gly	Phenylacetic Acid	phenylacetic acid	307.2	-29.5 ± 4.6
1295.204	Boc-L-Ile	Phenylacetic Acid	phenylacetic acid	363.2	-16.2 ± 8.9
1295.205	Boc-L-Leu	Phenylacetic Acid	phenylacetic acid	363.2	54.1 ± 17.5
1295.206	Boc-L-Val	Phenylacetic Acid	phenylacetic acid	349.2	32.7 ± 11.5
1295.207	Boc-L-Tyr(BrZ)	Phenylacetic Acid	phenylacetic acid	413.2	-85.0 ± 23.7
1295.208	Boc-D-Ala	Phenylacetic Acid	phenylacetic acid	321.2	-9.3 ± 14.3
1295.209	Boc-D-Phe	Phenylacetic Acid	phenylacetic acid	397.2	46.8 ± 19.0
1295.210	Boc-D-Ile	Phenylacetic Acid	phenylacetic acid	363.2	-17.7 ± 13.9
1295.211	Boc-D-Leu	Phenylacetic Acid	phenylacetic acid	363.2	38.8 ± 15.2
1295.212	Boc-D-Val	Phenylacetic Acid	phenylacetic acid	349.2	33.3 ± 15.5
1295.213	Boc-D-Tyr(BrZ)	Phenylacetic Acid	phenylacetic acid	413.2	-33.8 ± 29.9
1295.214	Boc-a-aminoisobutyric acid	Phenylacetic Acid	phenylacetic acid	335.2	1.6 ± 17.1
1295.215	Boc-L-Norvaline	Phenylacetic Acid	phenylacetic acid	349.2	31.5 ± 18.4
1295.216	Boc-D-Norvaline	Phenylacetic Acid	phenylacetic acid	349.2	24.5 ± 12.1
1295.217	Boc-L-Norleucine	Phenylacetic Acid	phenylacetic acid	363.2	13.0 ± 15.8
1295.218	Boc-D-Norleucine	Phenylacetic Acid	phenylacetic acid	363.2	-11.7 ± 2.2
1295.219	Boc-L-Phenylglycine	Phenylacetic Acid	phenylacetic acid	383.2	0.1 ± 2.8
1295.220	Boc-D-Phenylglycine	Phenylacetic Acid	phenylacetic acid	383.2	-5.2 ± 9.1
1295.221	Boc-L-Naphthylalanine	Phenylacetic Acid	phenylacetic acid	447.2	31.3 ± 2.1
1295.222	Boc-D-Naphthylalanine	Phenylacetic Acid	phenylacetic acid	447.2	22.4 ± 3.1
1295.223	Boc-L-cyclohexylAlanine	Phenylacetic Acid	phenylacetic acid	403.3	3.4 ± 0.9
1295.224	Boc-D-cyclohexylAlanine	Phenylacetic Acid	phenylacetic acid	403.3	9.6 ± 4.9
1295.225	Boc-L-4-Chlorophenylalanine	Phenylacetic Acid	phenylacetic acid	431.2	-32.6 ± 2.9
1295.226	Boc-D-4-Chlorophenylalanine	Phenylacetic Acid	phenylacetic acid	431.2	6.9 ± 1.9
1295.227	Boc-L-4-Fluorophenylalanine	Phenylacetic Acid	phenylacetic acid	415.2	-8.2 ± 7.0
1295.228	Boc-D-4-Fluorophenylalanine	Phenylacetic Acid	phenylacetic acid	415.2	31.3 ± 4.2
1295.229	Boc-L-(3-pyridyl)alanine	Phenylacetic Acid	phenylacetic acid	398.2	-23.1 ± 0.3
1295.230	Boc-D-(3-pyridyl)alanine	Phenylacetic Acid	phenylacetic acid	398.2	-16.0 ± 5.9
1295.231	Boc-L-a-tButylglycine	Phenylacetic Acid	phenylacetic acid	363.2	-31.5 ± 0.2
1295.232	Boc-D-a-tbutylglycine	Phenylacetic Acid	phenylacetic acid	363.2	8.2 ± 1.3
1295.233	Boc-O-ethyl-L-Tyrosine	Phenylacetic Acid	phenylacetic acid	441.2	31.0 ± 2.0
1295.234	Boc-O-ethyl-D-Tyrosine	Phenylacetic Acid	phenylacetic acid	441.2	-7.7 ± 1.0
1295.235	Boc-L-Phe	2-phenylbutyric acid	phenylacetic acid	425.3	35.8 ± 2.2
1295.236	Boc-L-Phe	3-Phenylbutyric Acid	phenylacetic acid	425.3	26.8 ± 8.6
1295.237	Boc-L-Phe	m-Tolylacetic Acid	phenylacetic acid	411.2	-4.8 ± 8.6
1295.238	Boc-L-Phe	3-Fluorophenylacetic Acid	phenylacetic acid	415.2	34.6 ± 10.8
1295.239	Boc-L-Phe	3-Bromophenylacetic Acid	phenylacetic acid	475.2	47.8 ± 4.4
1295.240	Boc-L-Phe	4-Fluorophenylacetic Acid	phenylacetic acid	415.2	52.2 ± 4.6
1295.241	Boc-L-Phe	3-Methoxyphenylacetic Acid	phenylacetic acid	427.2	7.4 ± 1.2
1295.242	Boc-L-Phe	4-Bromophenylacetic Acid	phenylacetic acid	475.2	67.1 ± 5.4
1295.243	Boc-L-Phe	4-Methoxyphenylacetic Acid	phenylacetic acid	427.2	27.2 ± 17.9
1295.244	Boc-L-Phe	4-Ethoxyphenylacetic Acid	phenylacetic acid	441.2	5.9 ± 3.0

1295.245	Boc-L-Phe	4-Isobutyl-alpha-Methylphenylacetic Acid	phenylacetic acid	467.3	21.5 ± 2.7
1295.246	Boc-L-Phe	3,5-Bis(Trifluoromethyl)-Phenylacetic Acid	phenylacetic acid	533.2	20.0 ± 0.0
1295.247	Boc-L-Phe	3-(3,4-Dimethoxyphenyl)-Propionic Acid	phenylacetic acid	471.3	32.5 ± 3.7
1295.248	Boc-L-Phe	4-Biphenylacetic Acid	phenylacetic acid	473.3	91.5 ± 4.0
1295.249	Boc-L-Phe	(3,4-Dimethoxyphenyl) Acetic Acid	phenylacetic acid	457.2	42.2 ± 4.4
1295.250	Boc-L-Phe	Phenylacetic Acid	phenylacetic acid	397.2	9.9 ± 2.3
1295.251	Boc-L-Phe	Hydrocinnamic Acid	phenylacetic acid	411.2	-112.4 ± 7.9
1295.252	Boc-L-Phe	4-Phenylbutyric Acid	phenylacetic acid	425.3	-2.3 ± 1.4
1295.253	Boc-L-Phe	Butyric Acid	phenylacetic acid	349.2	-12.1 ± 3.5
1295.254	Boc-L-Phe	Heptanoic Acid	phenylacetic acid	391.3	48.4 ± 2.8
1295.255	Boc-L-Phe	Isobutyric Acid	phenylacetic acid	349.2	-9.8 ± 2.4
1295.256	Boc-L-Phe	(+/-)-2-Methylbutyric Acid	phenylacetic acid	363.2	-8.1 ± 1.4
1295.257	Boc-L-Phe	Isovaleric Acid	phenylacetic acid	363.2	-28.5 ± 4.3
1295.258	Boc-L-Phe	4-Methylvaleric Acid	phenylacetic acid	377.3	-14.5 ± 5.2
1295.259	Boc-L-Phe	Trimethylacetic Acid	phenylacetic acid	363.2	-25.1 ± 3.1
1295.260	Boc-L-Phe	Cyclohexanecarboxylic Acid	phenylacetic acid	389.3	-10.7 ± 2.1
1295.261	Boc-L-Phe	Cyclohexylacetic Acid	phenylacetic acid	403.3	95.9 ± 0.0
1295.262	Boc-L-Phe	Cyclohexanebutyric Acid	phenylacetic acid	431.3	-30.5 ± 6.8
1295.263	Boc-L-Phe	Cycloheptanecarboxylic Acid	phenylacetic acid	403.3	-8.4 ± 3.8
1295.264	Boc-L-Phe	Acetic Acid	phenylacetic acid	321.2	7.5 ± 1.6
1295.265	Boc-L-Phe	Cyclobutanecarboxylic Acid	phenylacetic acid	361.2	53.1 ± 5.5
1295.266	Boc-L-Phe	Cyclopentanecarboxylic Acid	phenylacetic acid	375.2	99.6 ± 0.2
1295.267	Boc-L-Phe	3-Cyclopentylpropionic Acid	phenylacetic acid	403.3	56.2 ± 9.5
1295.268	Boc-L-Phe	Cyclohexanepropionic Acid	phenylacetic acid	417.3	103.1 ± 0.2
1295.269	Boc-L-Phe	4-Methyl-1-Cyclohexanecarboxylic Acid	phenylacetic acid	403.3	104.2 ± 0.1
1295.270	Boc-L-Phe	2-Norbornaneacetic Acid	phenylacetic acid	415.3	84.6 ± 8.0
1295.271	Boc-L-Phe	1-Adamantaneacetic Acid	phenylacetic acid	455.3	36.0 ± 17.8
1295.272	Boc-L-Phe	Phenylacetic Acid	1-phenyl-1cyclopropanecarboxylic acid	423.2	96.0 ± 1.7
1295.273	Boc-L-Phe	Phenylacetic Acid	2-Phenylbutyric Acid	425.3	104.4 ± 1.1
1295.274	Boc-L-Phe	Phenylacetic Acid	3-Phenylbutyric Acid	425.3	104.9 ± 1.1
1295.275	Boc-L-Phe	Phenylacetic Acid	m-Tolylacetic Acid	411.2	60.7 ± 6.4
1295.276	Boc-L-Phe	Phenylacetic Acid	3-Fluorophenylacetic Acid	415.2	104.2 ± 1.8
1295.277	Boc-L-Phe	Phenylacetic Acid	3-Bromophenylacetic Acid	475.2	97.6 ± 2.7
1295.278	Boc-L-Phe	Phenylacetic Acid	4-Fluorophenylacetic Acid	415.2	29.8 ± 16.2
1295.279	Boc-L-Phe	Phenylacetic Acid	3-Methoxyphenylacetic Acid	427.2	75.2 ± 8.9
1295.280	Boc-L-Phe	Phenylacetic Acid	4-Bromophenylacetic Acid	475.2	42.0 ± 6.0
1295.281	Boc-L-Phe	Phenylacetic Acid	4-Methoxyphenylacetic Acid	427.2	52.6 ± 3.9
1295.282	Boc-L-Phe	Phenylacetic Acid	4-Ethoxyphenylacetic Acid	441.2	-2.3 ± 4.3
1295.283	Boc-L-Phe	Phenylacetic Acid	4-Isobutyl-alpha-Methylphenylacetic Acid	467.3	58.6 ± 1.4

1295.284	Boc-L-Phe	Phenylacetic Acid	3-(3,4-Dimethoxyphenyl)-Propionic Acid	471.3	34.0	± 5.0
1295.285	Boc-L-Phe	Phenylacetic Acid	4-Biphenylacetic Acid	473.3	11.1	± 1.0
1295.286	Boc-L-Phe	Phenylacetic Acid	2-(Trifluoromethyl)-cinnamic Acid	477.2	91.9	± 0.9
1295.287	Boc-L-Phe	Phenylacetic Acid	(3,4-Dimethoxyphenyl) Acetic Acid	457.2	54.7	± 1.2
1295.288	Boc-L-Phe	Phenylacetic Acid	m-toluic acid	397.2	59.9	± 2.5
1295.289	Boc-L-Phe	Phenylacetic Acid	Hydrocinnamic Acid	411.2	83.1	± 6.9
1295.290	Boc-L-Phe	Phenylacetic Acid	4-phenylbutyric acid	425.3	60.2	± 7.8
1295.291	Boc-L-Phe	Phenylacetic Acid	phenylacetic acid	397.2	5.6	± 0.7
1295.292	Boc-L-Phe	Phenylacetic Acid	4-Ethyl-4-Biphenylcarboxylic Acid	487.3	48.3	± 2.5
1295.293	Boc-L-Phe	Phenylacetic Acid	Butyric Acid	349.2	37.4	± 0.8
1295.294	Boc-L-Phe	Phenylacetic Acid	Heptanoic Acid	391.3	44.3	± 0.4
1295.295	Boc-L-Phe	Phenylacetic Acid	Isobutyric Acid	349.2	31.3	± 4.6
1295.296	Boc-L-Phe	Phenylacetic Acid	(+/-)-2-Methylbutyric Acid	363.2	41.7	± 1.5
1295.297	Boc-L-Phe	Phenylacetic Acid	4-Methylvaleric Acid	377.3	62.2	± 3.6
1295.298	Boc-L-Phe	Phenylacetic Acid	Trimethylacetic Acid	363.2	94.7	± 1.6
1295.299	Boc-L-Phe	Phenylacetic Acid	Cyclohexylacetic Acid	403.3	89.6	± 1.1
1295.300	Boc-L-Phe	Phenylacetic Acid	Cyclohexanebutyric Acid	431.3	82.8	± 5.4
1295.301	Boc-L-Phe	Phenylacetic Acid	Acetic Acid	321.2	15.4	± 1.7
1295.302	Boc-L-Phe	Phenylacetic Acid	Cyclobutanecarboxylic Acid	361.2	24.7	± 0.1
1295.303	Boc-L-Phe	Phenylacetic Acid	Cyclopentanecarboxylic Acid	375.2	3.3	± 2.0
1295.304	Boc-L-Phe	Phenylacetic Acid	3-Cyclopentylpropionic Acid	403.3	45.5	± 2.6
1295.305	Boc-L-Phe	Phenylacetic Acid	Cyclohexanepropionic Acid	417.3	82.8	± 1.8
1295.306	Boc-L-Phe	Phenylacetic Acid	4-Methyl-1-Cyclohexanecarboxylic Acid	403.3	97.8	± 1.3
1295.307	Boc-L-Phe	Phenylacetic Acid	2-Norbornaneacetic Acid	415.3	100.6	± 0.3

Table S3: Building blocks used for the preparation of individual componds **2520**

Bag #	**Building block used for R1**	**Building block used for R2**	**Building block used for R3**
1	Boc-Ala(2-naphtyl)-OH	3-PhenylButyric Acid	2-cyclopentylpropioinic acid
2	Boc-Ala(2-naphtyl)-OH	2-Norbaneacetic acid	4-methoxyphenylacetic acid
3	Boc-Ala(2-naphtyl)-OH	cyclohexanecarboxylic acid	2-phenylbutyric acid
4	Boc-Ala(2-naphtyl)-OH	3-PhenylButyric Acid	isobutyric acid
5	Boc-Ala(2-naphtyl)-OH	2-Norbaneacetic acid	2-cyclopentylpropioinic acid
6	Boc-Ala(2-naphtyl)-OH	cyclohexanecarboxylic acid	4-methoxyphenylacetic acid
7	Boc-Ala(2-naphtyl)-OH	3-PhenylButyric Acid	2-phenylbutyric acid
8	Boc-Ala(2-naphtyl)-OH	2-Norbaneacetic acid	isobutyric acid
9	Boc-Ala(2-naphtyl)-OH	cyclohexanecarboxylic acid	2-cyclopentylpropioinic acid
10	Boc-Ala(2-naphtyl)-OH	3-PhenylButyric Acid	4-methoxyphenylacetic acid
11	Boc-Ala(2-naphtyl)-OH	2-Norbaneacetic acid	2-phenylbutyric acid
12	Boc-Ala(2-naphtyl)-OH	cyclohexanecarboxylic acid	isobutyric acid
13	Boc-L-Isoluceine-OH	3-PhenylButyric Acid	2-cyclopentylpropioinic acid
14	Boc-L-Isoluceine-OH	2-Norbaneacetic acid	4-methoxyphenylacetic acid
15	Boc-L-Isoluceine-OH	cyclohexanecarboxylic acid	2-phenylbutyric acid
16	Boc-L-Isoluceine-OH	3-PhenylButyric Acid	isobutyric acid

17	Boc-L-Isoluceine-OH	2-Norbaneacetic acid	2-cyclopentylpropioinic acid
18	Boc-L-Isoluceine-OH	cyclohexanecarboxylic acid	4-methoxyphenylacetic acid
19	Boc-L-Isoluceine-OH	3-PhenylButyric Acid	2-phenylbutyric acid
20	Boc-L-Isoluceine-OH	2-Norbaneacetic acid	isobutyric acid
21	Boc-L-Isoluceine-OH	cyclohexanecarboxylic acid	2-cyclopentylpropioinic acid
22	Boc-L-Isoluceine-OH	3-PhenylButyric Acid	4-methoxyphenylacetic acid
23	Boc-L-Isoluceine-OH	2-Norbaneacetic acid	2-phenylbutyric acid
24	Boc-L-Isoluceine-OH	cyclohexanecarboxylic acid	isobutyric acid
25	Boc-L-Phe-OH	3-PhenylButyric Acid	2-cyclopentylpropioinic acid
26	Boc-L-Phe-OH	2-Norbaneacetic acid	4-methoxyphenylacetic acid
27	Boc-L-Phe-OH	cyclohexanecarboxylic acid	2-phenylbutyric acid
28	Boc-L-Phe-OH	3-PhenylButyric Acid	isobutyric acid
29	Boc-L-Phe-OH	2-Norbaneacetic acid	2-cyclopentylpropioinic acid
30	Boc-L-Phe-OH	cyclohexanecarboxylic acid	4-methoxyphenylacetic acid
31	Boc-L-Phe-OH	3-PhenylButyric Acid	2-phenylbutyric acid
32	Boc-L-Phe-OH	2-Norbaneacetic acid	isobutyric acid
33	Boc-L-Phe-OH	cyclohexanecarboxylic acid	2-cyclopentylpropioinic acid
34	Boc-L-Phe-OH	3-PhenylButyric Acid	4-methoxyphenylacetic acid
35	Boc-L-Phe-OH	2-Norbaneacetic acid	2-phenylbutyric acid
36	Boc-L-Phe-OH	cyclohexanecarboxylic acid	isobutyric acid
37	Boc-L-Tyr(Et)-OH	3-PhenylButyric Acid	2-cyclopentylpropioinic acid
38	Boc-L-Tyr(Et)-OH	2-Norbaneacetic acid	4-methoxyphenylacetic acid
39	Boc-L-Tyr(Et)-OH	cyclohexanecarboxylic acid	2-phenylbutyric acid
40	Boc-L-Tyr(Et)-OH	3-PhenylButyric Acid	isobutyric acid
41	Boc-L-Tyr(Et)-OH	2-Norbaneacetic acid	2-cyclopentylpropioinic acid
42	Boc-L-Tyr(Et)-OH	cyclohexanecarboxylic acid	4-methoxyphenylacetic acid
43	Boc-L-Tyr(Et)-OH	3-PhenylButyric Acid	2-phenylbutyric acid
44	Boc-L-Tyr(Et)-OH	2-Norbaneacetic acid	isobutyric acid
45	Boc-L-Tyr(Et)-OH	cyclohexanecarboxylic acid	2-cyclopentylpropioinic acid
46	Boc-L-Tyr(Et)-OH	3-PhenylButyric Acid	4-methoxyphenylacetic acid
47	Boc-L-Tyr(Et)-OH	2-Norbaneacetic acid	2-phenylbutyric acid
48	Boc-L-Tyr(Et)-OH	cyclohexanecarboxylic acid	isobutyric acid

Activity of Library 2520: deconvolution of FIU-1295

	% Inhibition at 4 μM (N=3)	IC_{50} (μM, N=4) ROR-Gamma	ROR-Beta	ROR-Alpha
2520.01	40.6 ± 7.4			
2520.02	-33.8 ± 2.8			
2520.03	-31.8 ± 7.3			
2520.04	-40.8 ± 1.1			
2520.05	43.1 ± 11.1			
2520.06	-56.4 ± 1.5			
2520.07	-32.2 ± 14.0			
2520.08	-23.6 ± 11.3			
2520.09	-25.4 ± 11.4			
2520.10	-7.3 ± 1.8			

2520.11	67.6 ± 3.3	7.55 ± 0.5	7.3 ± 0.8	10.3 ± 0.4
2520.12	30.4 ± 4.0			
2520.13	8.8 ± 0.7			
2520.14	43.0 ± 6.2			
2520.15	43.6 ± 11.4	4.69 ± 0.2	3.66 ± 0.3	5.97 ± 0.2
2520.16	7.1 ± 16.3			
2520.17	31.7 ± 0.7			
2520.18	26.2 ± 4.1			
2520.19	75.9 ± 8.9	5.07 ± 0.3	4.87 ± 0.3	6.74 ± 0.3
2520.20	13.3 ± 10.4			
2520.21	37.9 ± 5.2			
2520.22	10.1 ± 13.3			
2520.23	35.9 ± 13.1			
2520.24	-23.6 ± 2.6			
2520.25	-6.3 ± 2.8			
2520.26	40.8 ± 1.1			
2520.27	97.7 ± 1.3	4.8 ± 0.2	4.89 ± 0.3	6.61 ± 0.3
2520.28	69.8 ± 1.9	6.37 ± 0.4	6.17 ± 0.3	8.48 ± 0.4
2520.29	45.8 ± 9.4			
2520.30	-4.8 ± 8.8			
2520.31	70.5 ± 9.2	3.95 ± 0.3	5.5 ± 0.3	6.82 ± 0.4
2520.32	36.0 ± 17.5			
2520.33	61.1 ± 4.9	6.27 ± 0.3	6.19 ± 0.4	7.27 ± 0.3
2520.34	34.4 ± 6.2			
2520.35	-21.7 ± 6.6			
2520.36	-5.4 ± 5.2			
2520.37	-37.4 ± 18.9			
2520.38	-22.6 ± 11.9			
2520.39	73.2 ± 3.0	4.36 ± 0.2	5.34 ± 0.4	6.36 ± 0.3
2520.40	-8.1 ± 6.6			
2520.41	-13.4 ± 7.7			
2520.42	96.5 ± 0.9	5.17 ± 0.8	5.21 ± 0.3	5.4 ± 0.4
2520.43	-5.4 ± 2.1			
2520.44	-20.0 ± 9.6			
2520.45	67.6 ± 8.3	5.33 ± 0.2	5.51 ± 0.3	5.86 ± 0.3
2520.46	67.3 ± 2.9	5.31 ± 0.2	5.46 ± 0.3	5.82 ± 0.3
2520.47	51.3 ± 9.7	4.68 ± 0.2	6.67 ± 0.4	7.20 ± 0.4
2520.48	69.2 ± 32.0	9.27 ± 0.4	6.57 ± 0.5	9.61 ± 0.5

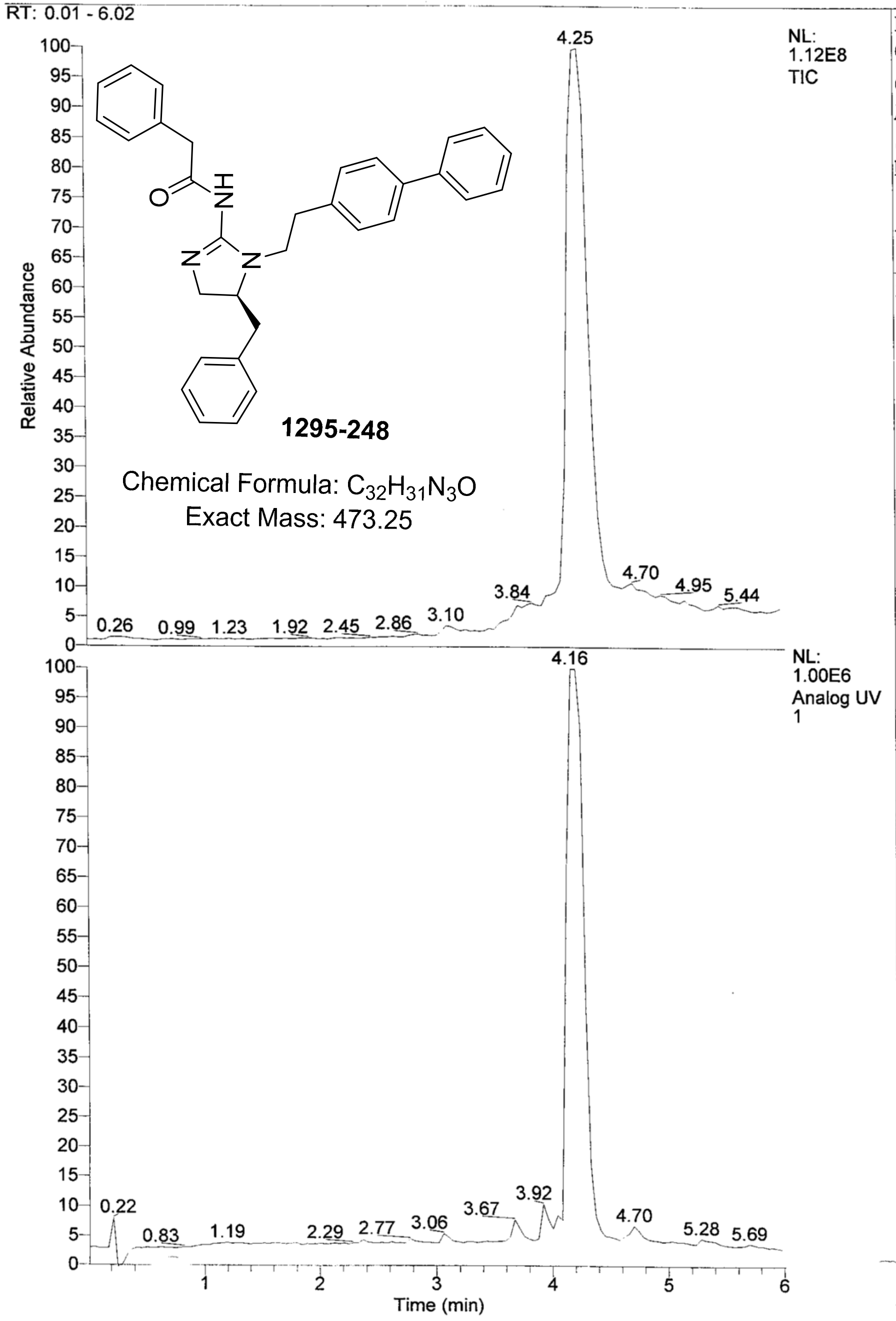


```
Experiment Method:
C:\LCQ\Methods\ESI\Short-col\ESI5-95m8  Created: 11/11/97
Creator: LCQ                          Last modified: 3/2/01 by
Administrator
Summary: 5-95m8
MS Run Time (min): 6.00

Autosampler Settings:
```

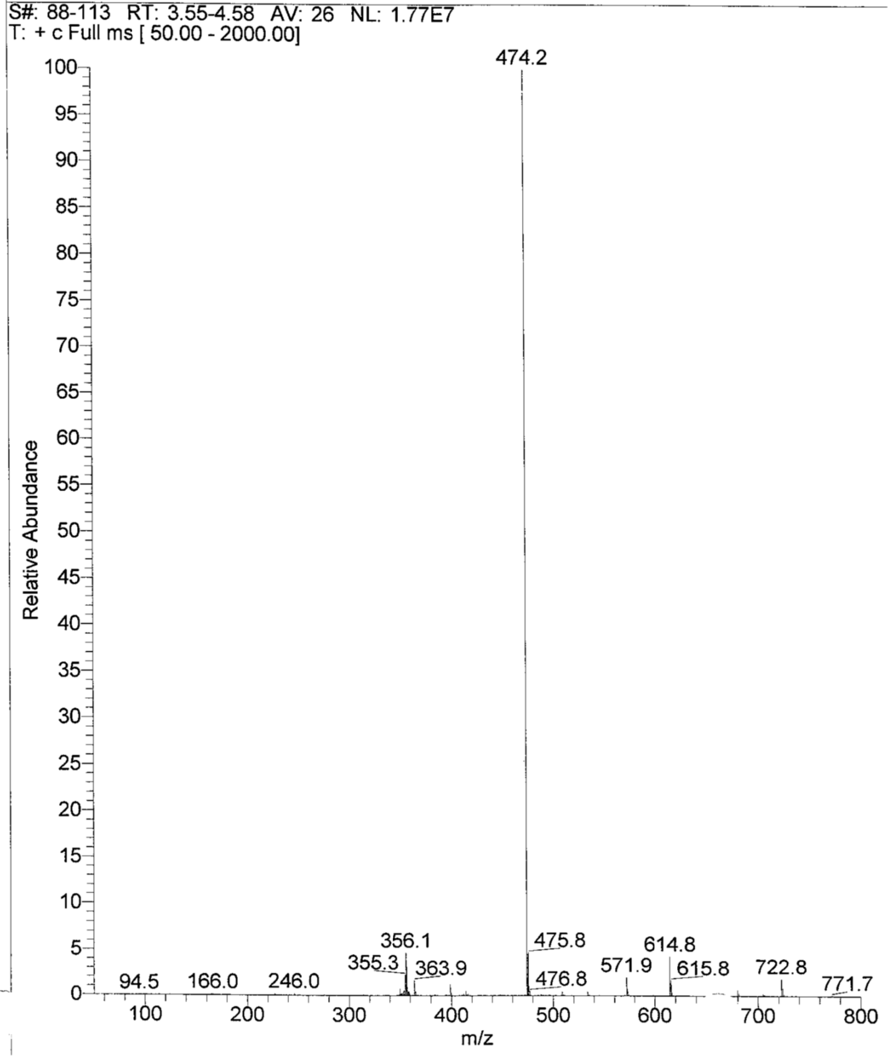

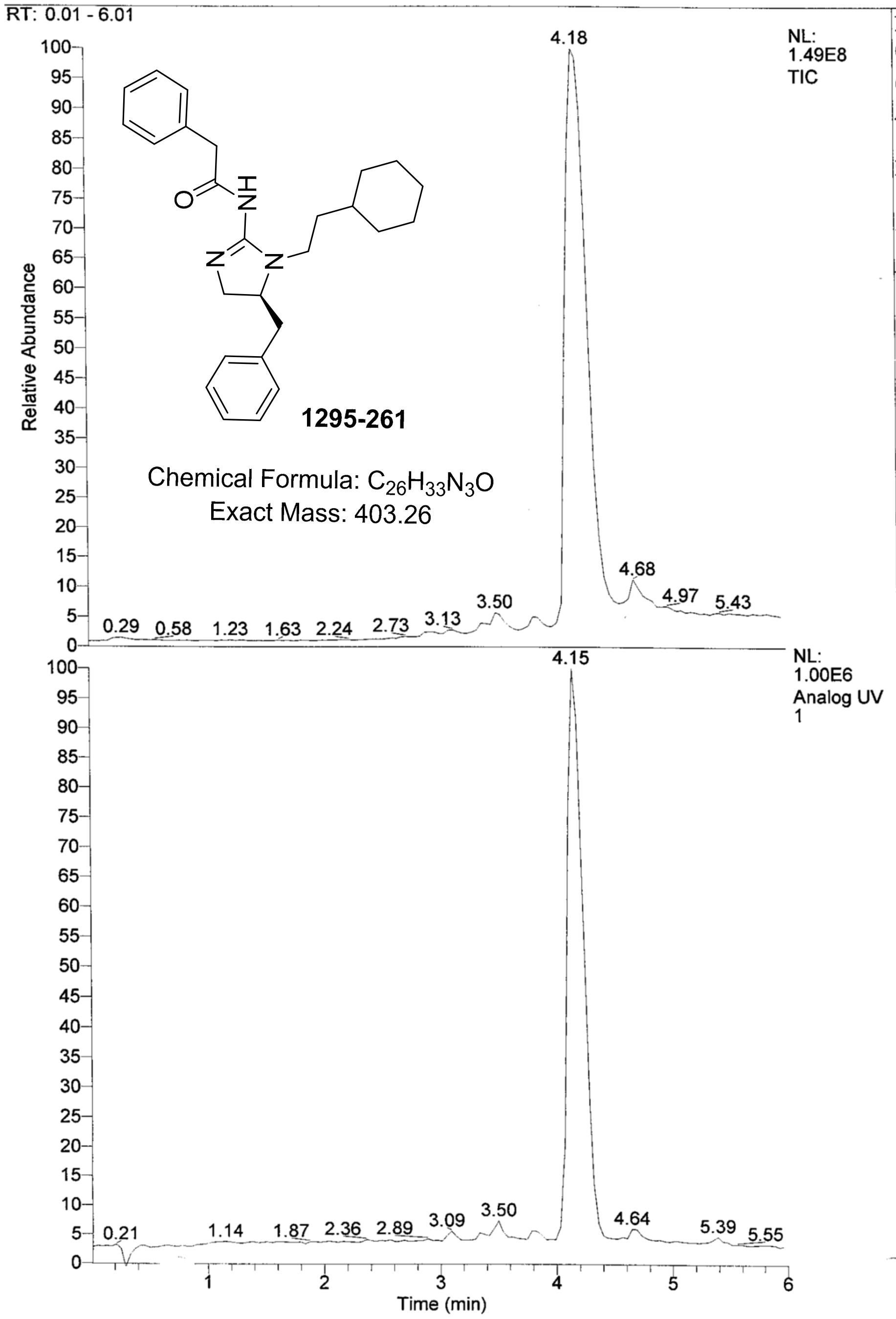

1295-261

Chemical Formula: $C_{26}H_{33}N_3O$
Exact Mass: 403.26

Experiment Method:
C:\LCQ\Methods\ESI\Short-col\ESI5-95m8 Created: 11/11/97
Creator: LCQ Last modified: 3/3/01 by
Administrator
Summary: 5-95m8
MS Run Time (min): 6.00

Autosampler Settings:

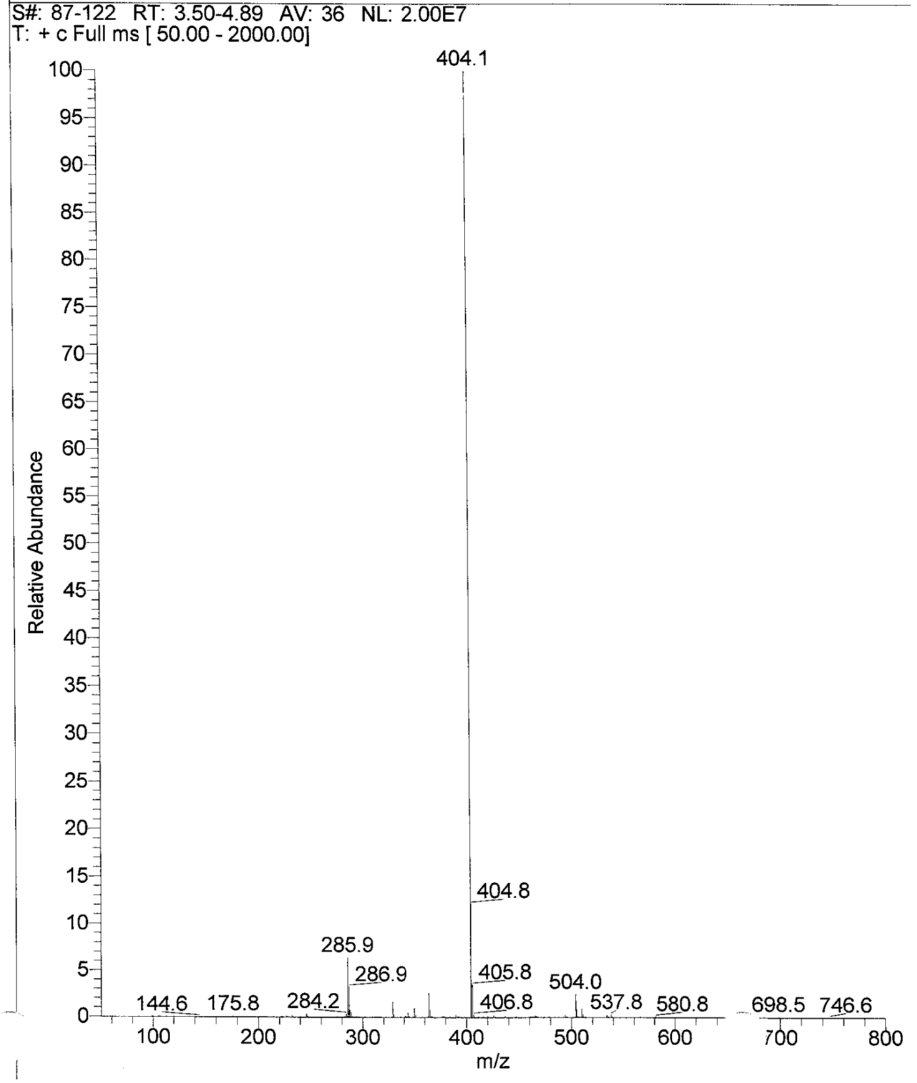

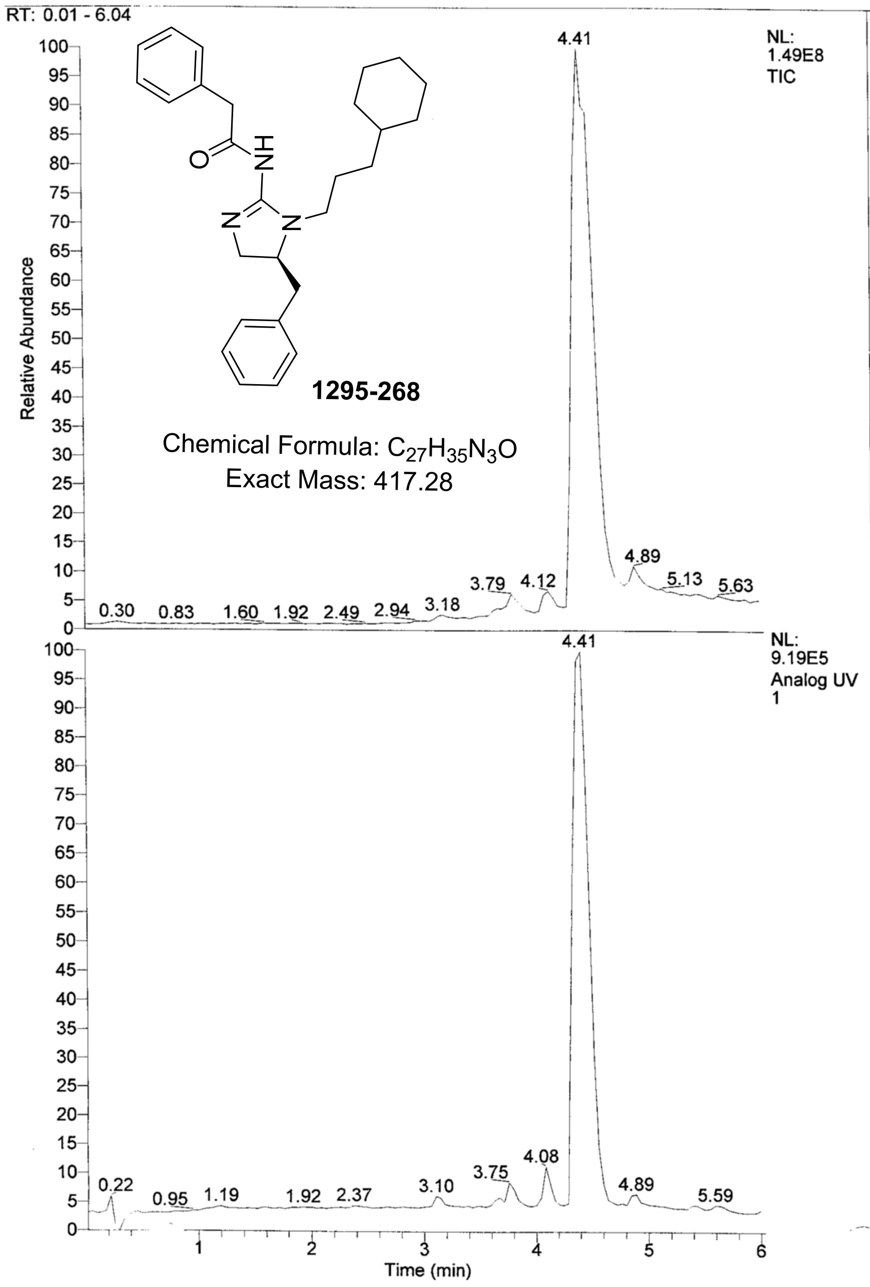

Experiment Method:
C:\LCQ\Methods\ESI\Short-col\ESI5-95m8 Created: 11/11/97
Creator: LCQ Last modified: 3/3/01 by Administrator
Summary: 5-95m8
MS Run Time (min): 6.00

Autosampler Settings:

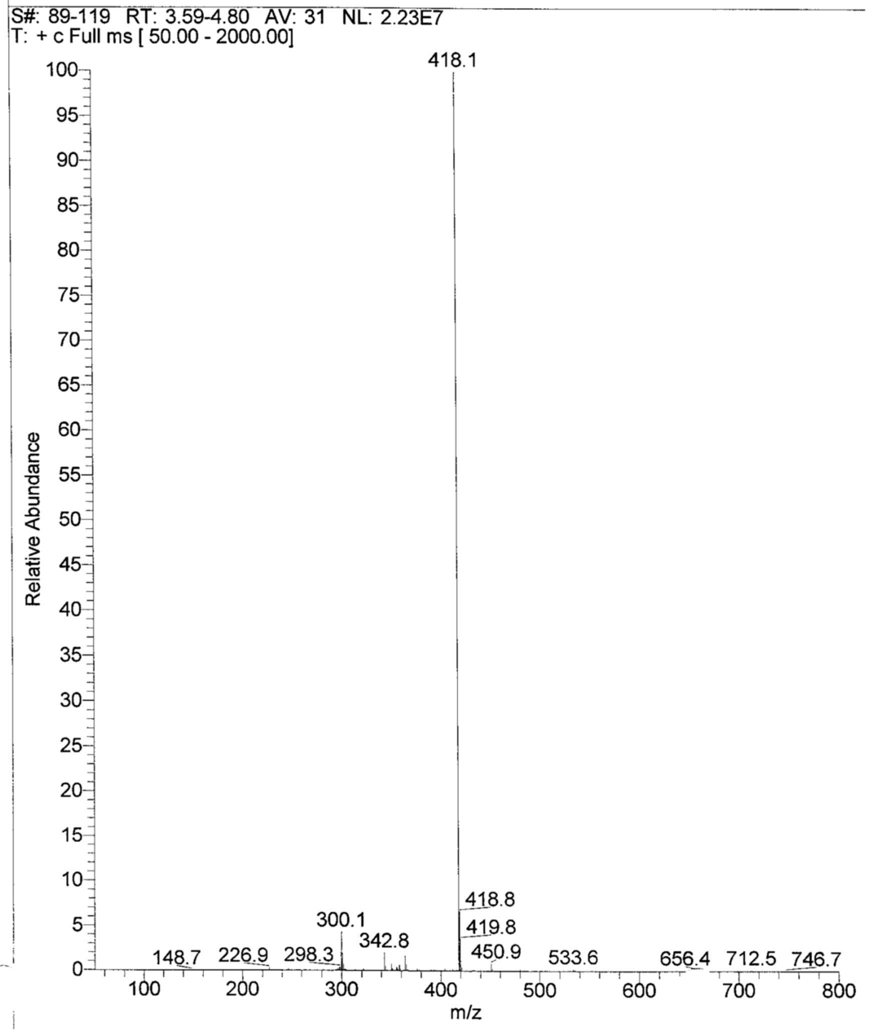

1295-269

Chemical Formula: $C_{26}H_{33}N_3O$
Exact Mass: 403.26

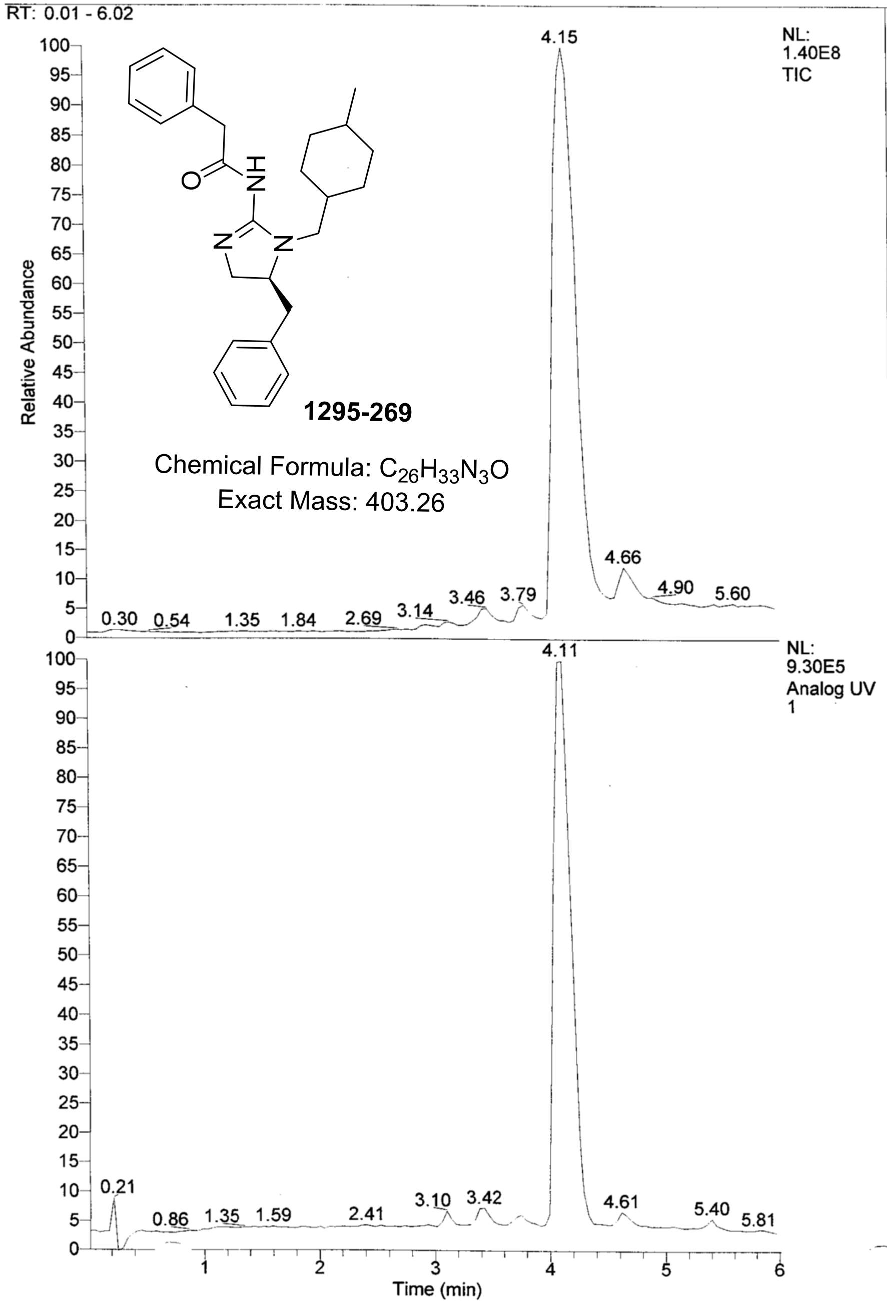

Experiment Method:
C:\LCQ\Methods\ESI\Short-col\ESI5-95m8 Created: 11/11/97
Creator: LCQ Last modified: 3/3/01 by
Administrator
Summary: 5-95m8
MS Run Time (min): 6.00

Autosampler Settings:

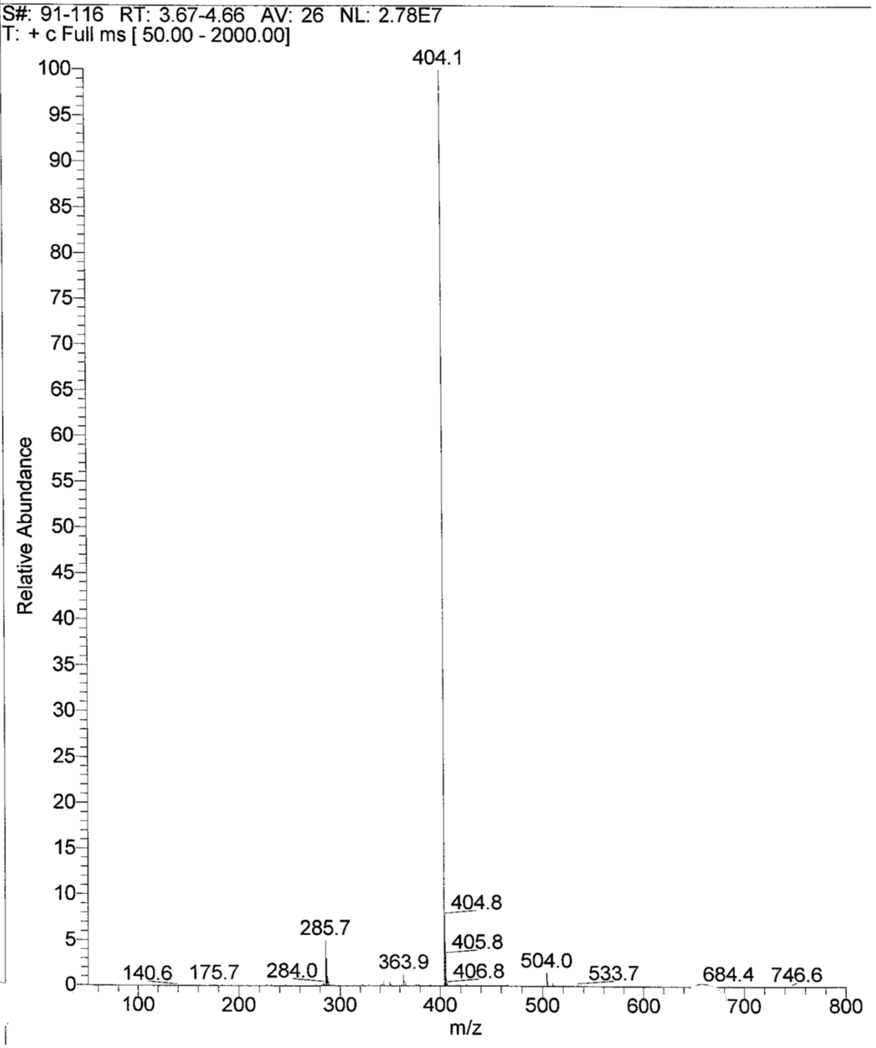

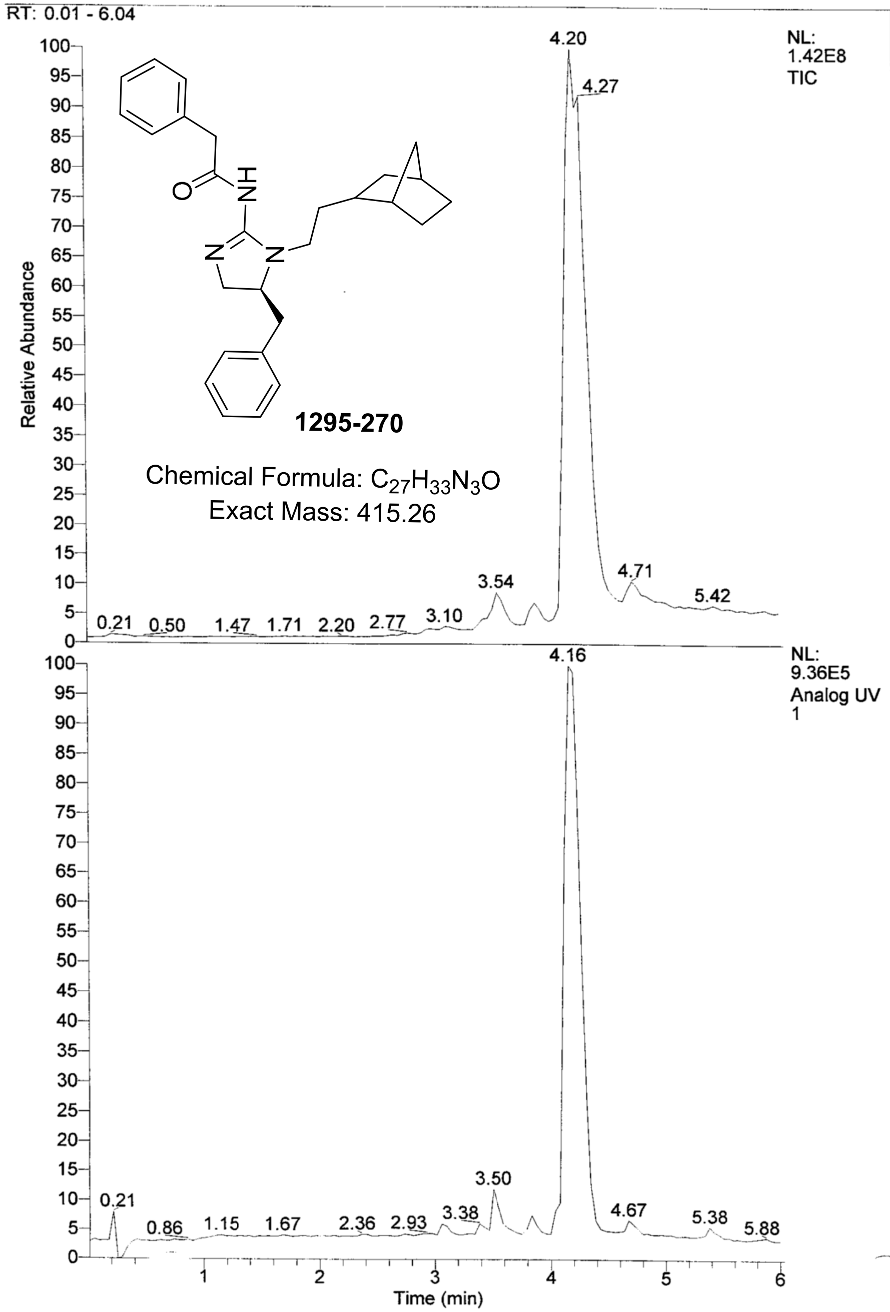

Experiment Method:
C:\LCQ\Methods\ESI\Short-col\ESI5-95m8 Created: 11/11/97
Creator: LCQ Last modified: 3/3/01 by
Administrator
Summary: 5-95m8
MS Run Time (min): 6.00

Autosampler Settings:

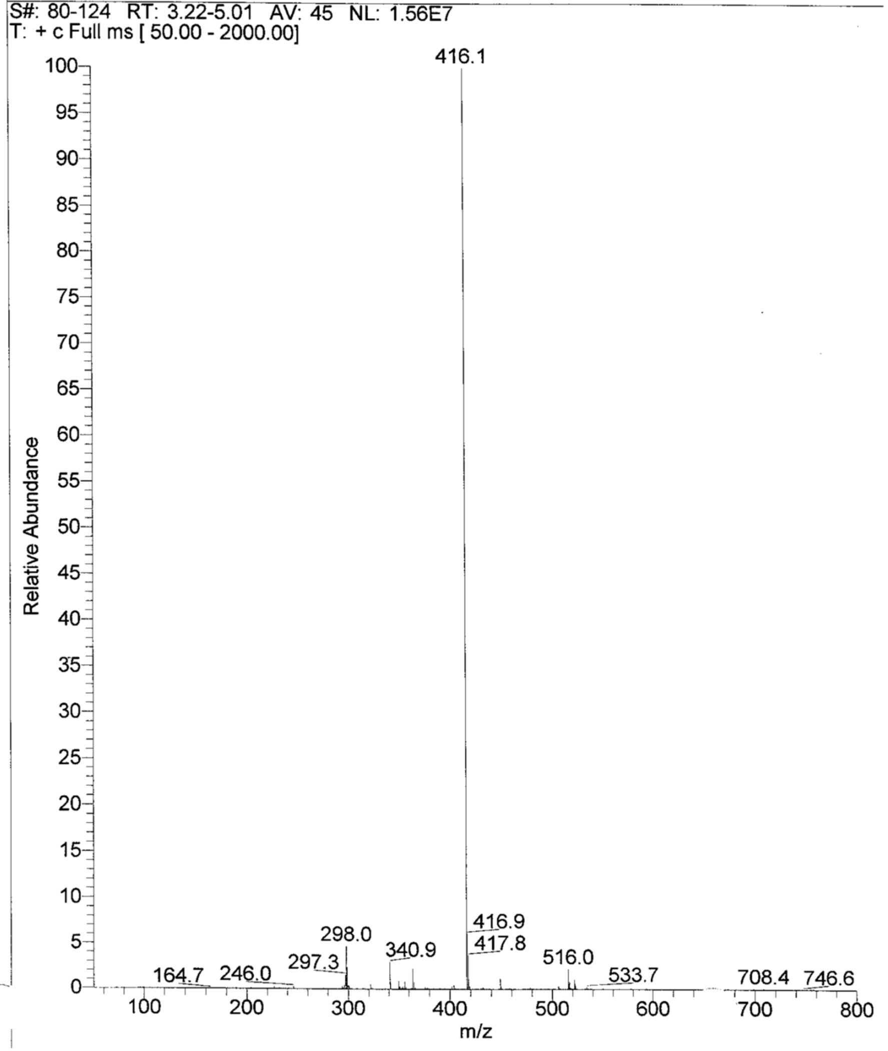

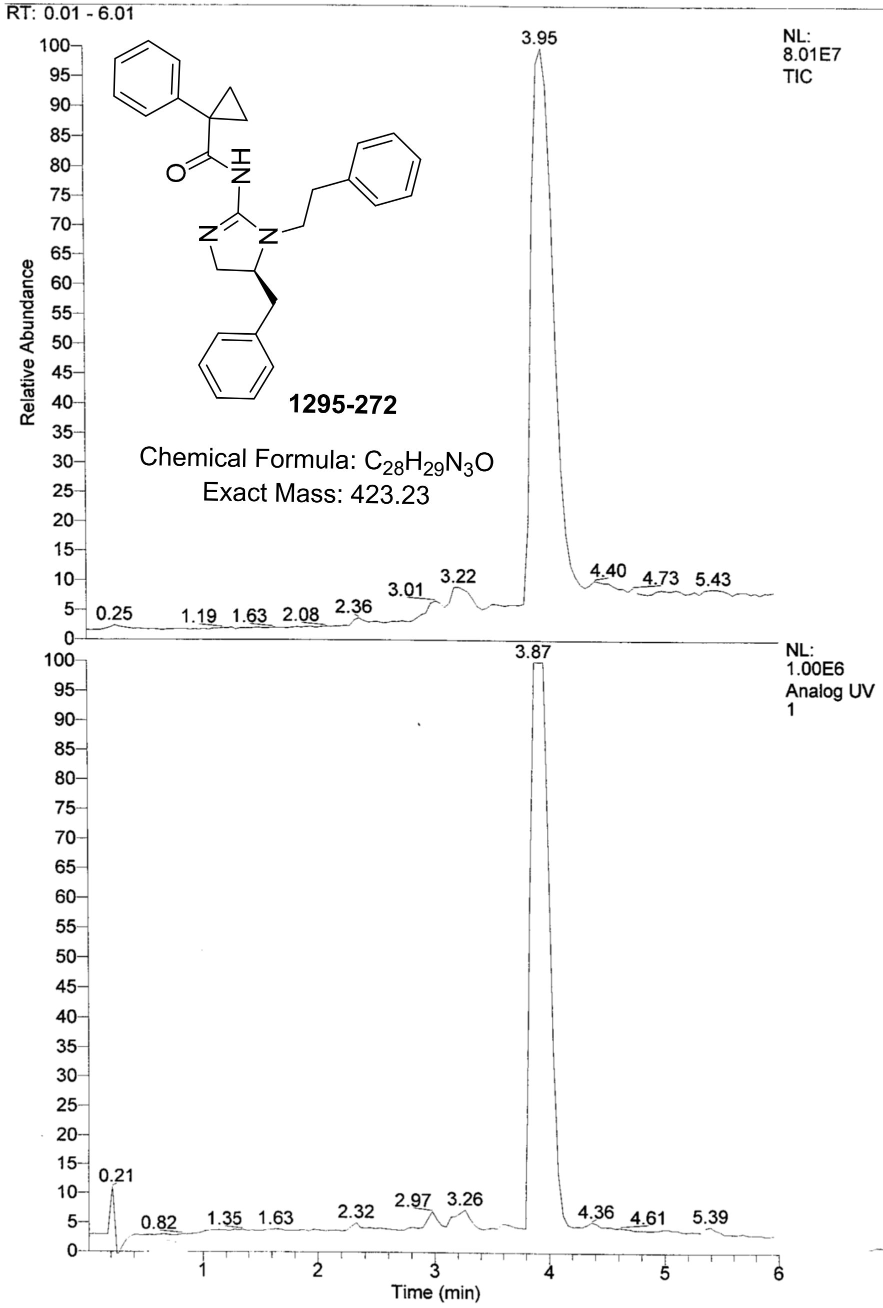

Experiment Method:
C:\LCQ\Methods\ESI\Short-col\ESI5-95m8 Created: 11/11/97
Creator: LCQ Last modified: 3/3/01 by
Administrator
Summary: 5-95m8
MS Run Time (min): 6.00

Autosampler Settings:

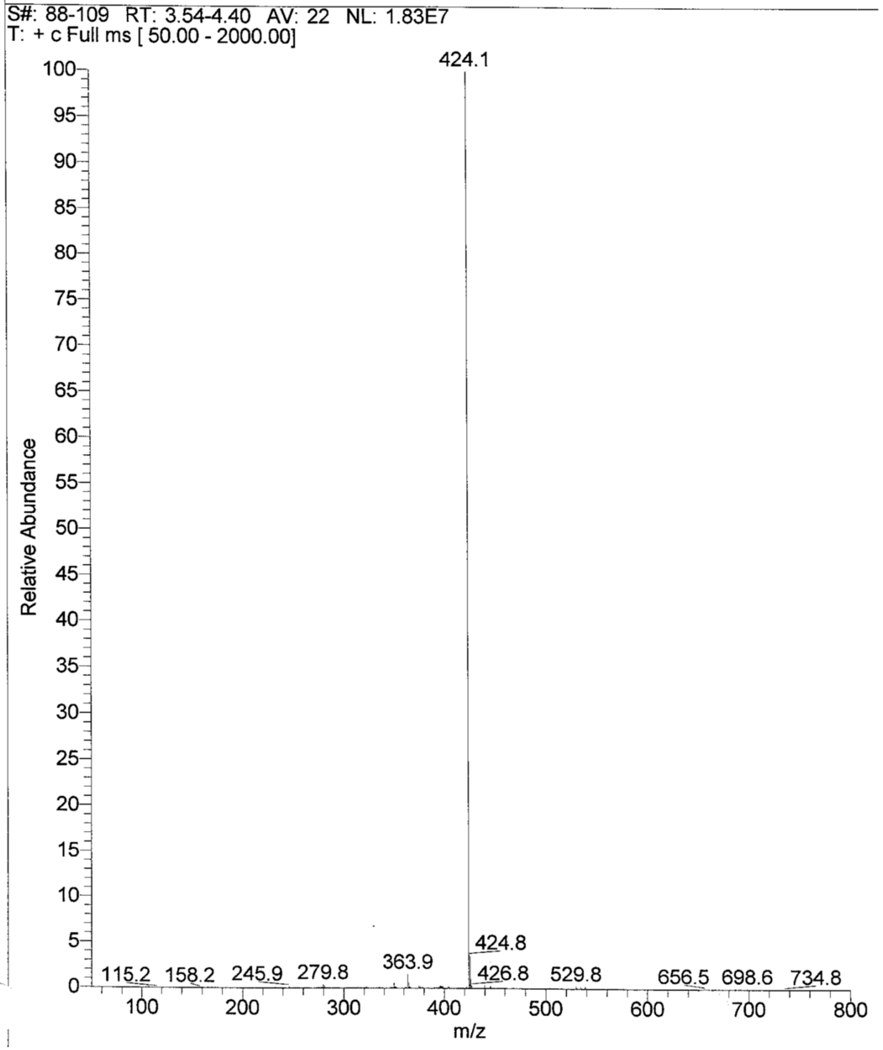

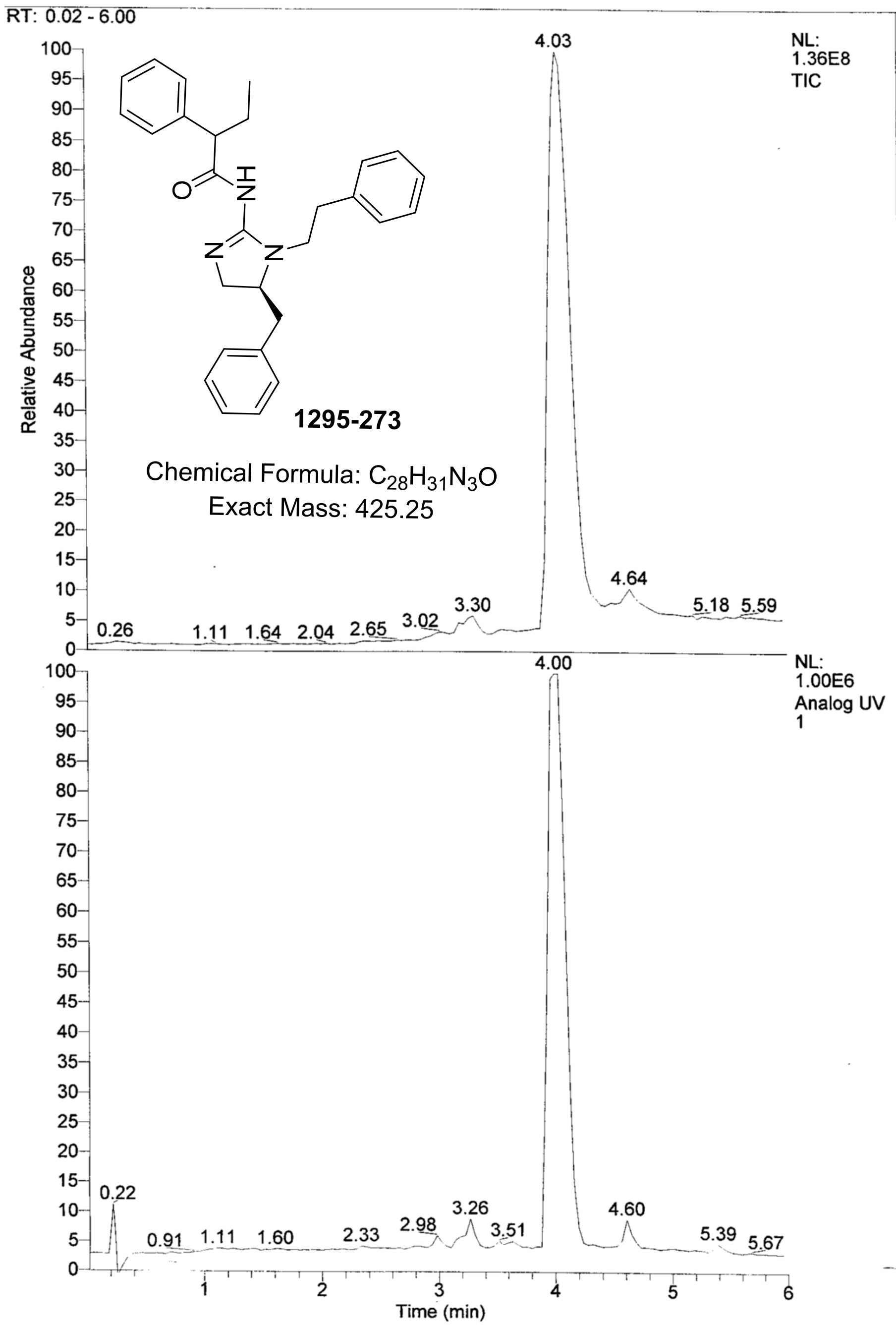

1295-273

Chemical Formula: $C_{28}H_{31}N_3O$
Exact Mass: 425.25

Experiment Method:
C:\LCQ\Methods\ESI\Short-col\ESI5-95m8 Created: 11/11/97
Creator: LCQ Last modified: 3/3/01 by
Administrator
Summary: 5-95m8
MS Run Time (min): 6.00

Autosampler Settings:

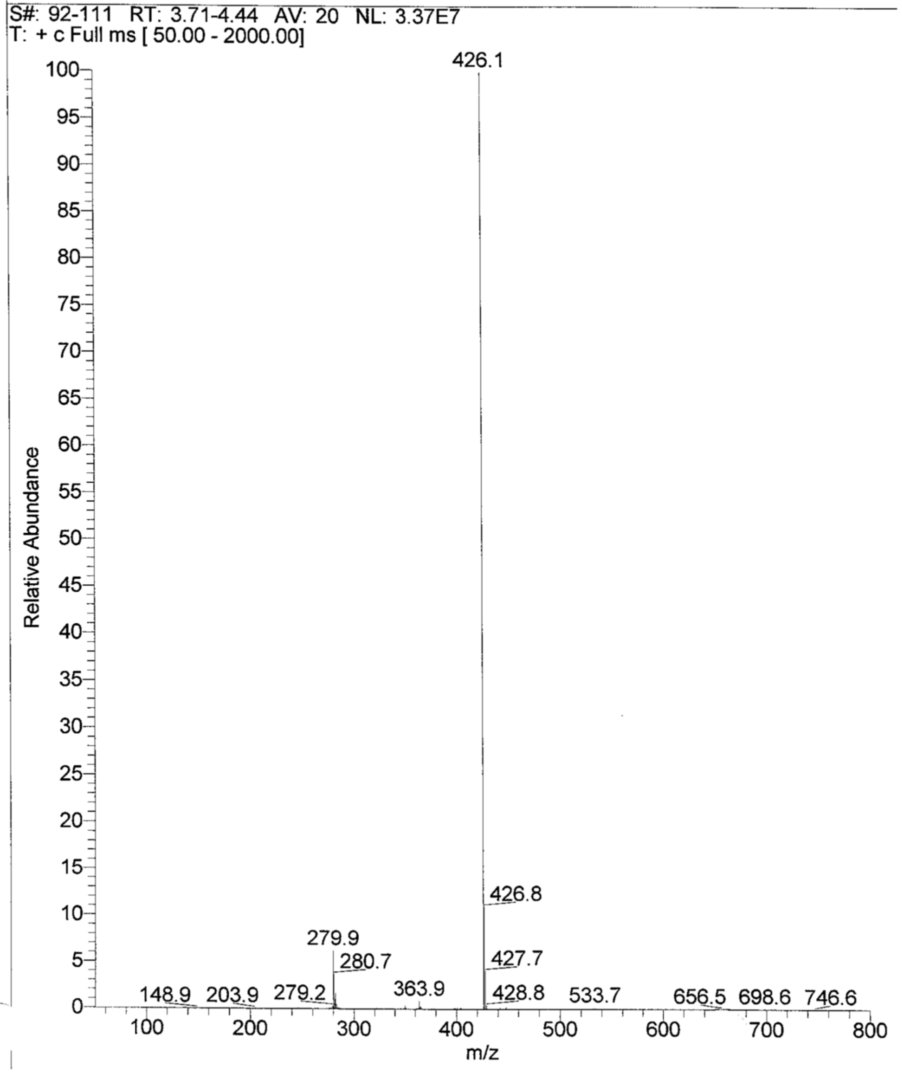

1295-274

Chemical Formula: $C_{28}H_{31}N_3O$

Exact Mass: 425.25

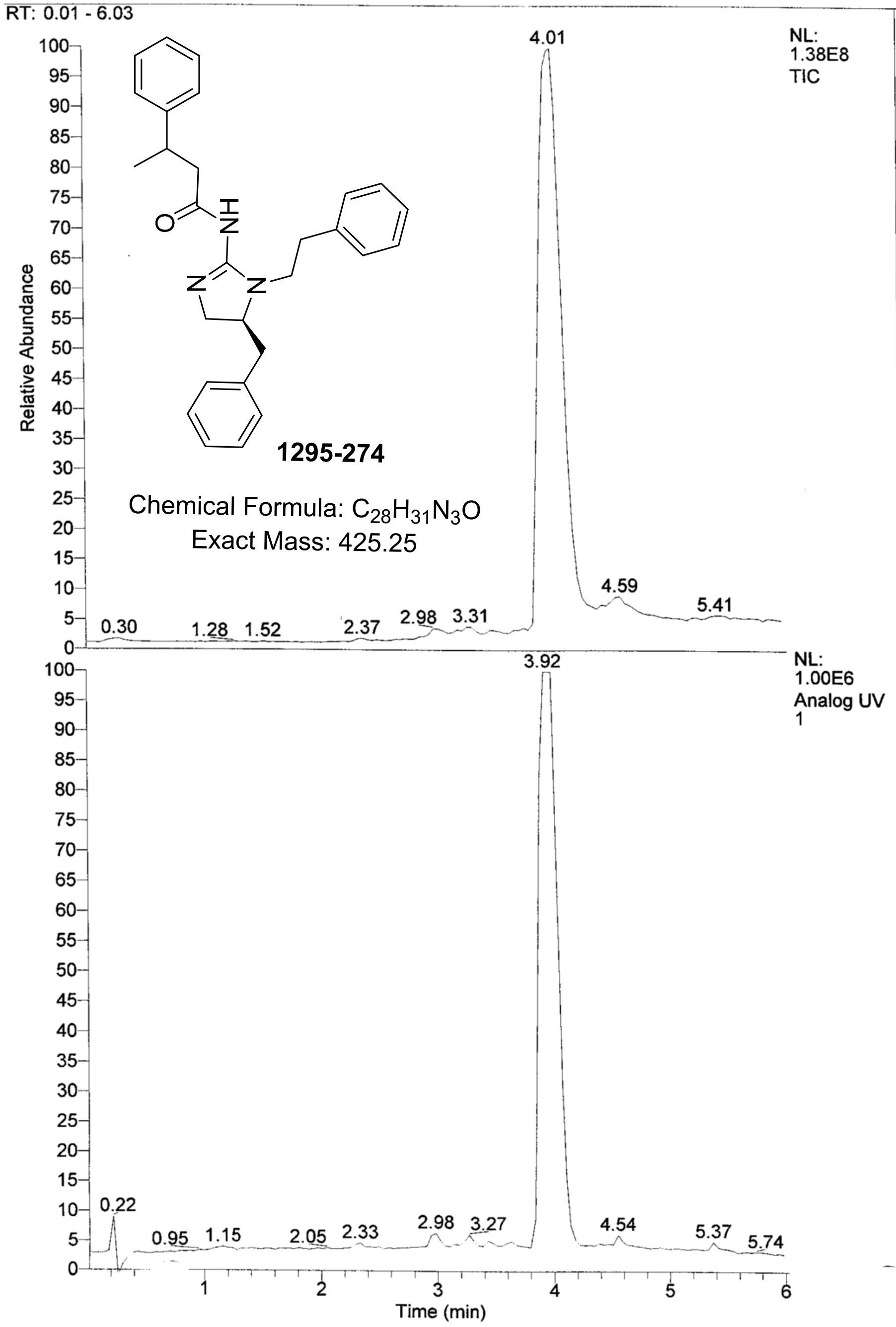

Experiment Method:
C:\LCQ\Methods\ESI\Short-col\ESI5-95m8 Created: 11/11/97
Creator: LCQ Last modified: 3/3/01 by Administrator
Summary: 5-95m8
MS Run Time (min): 6.00

Autosampler Settings:

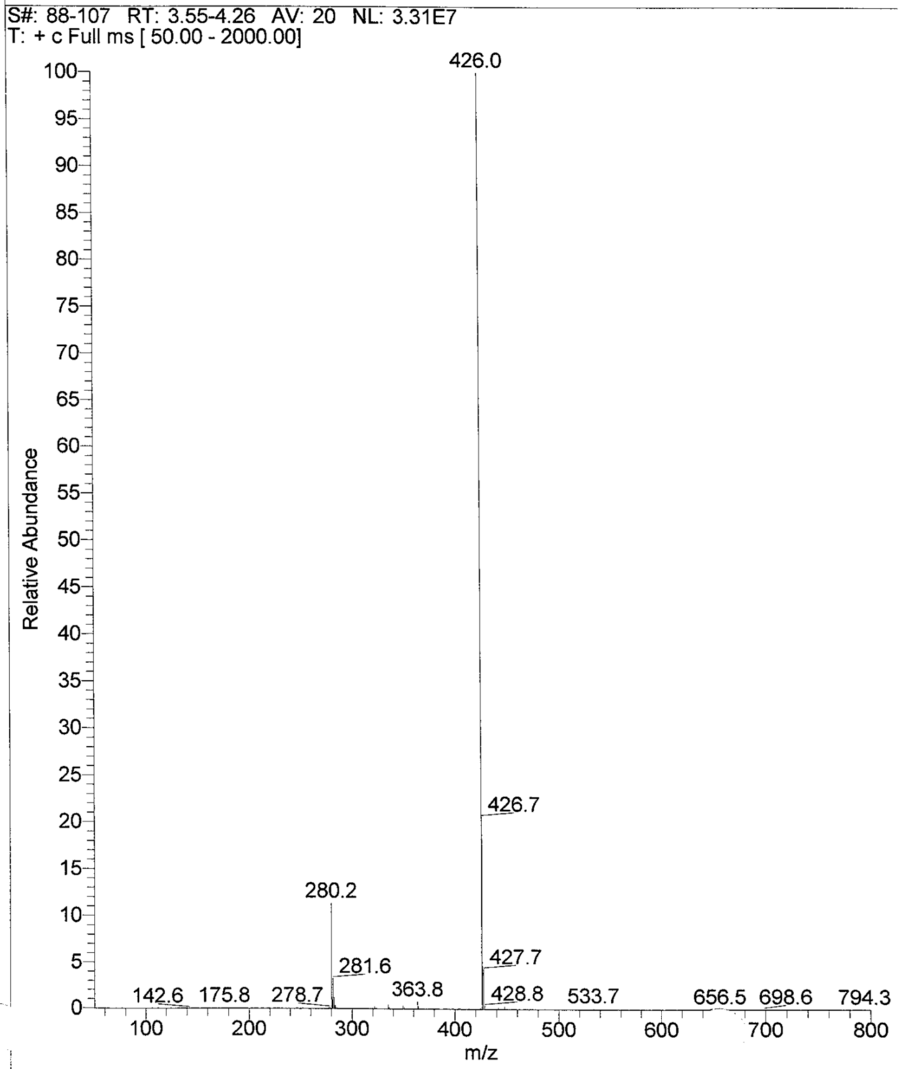

1295-289

Chemical Formula: $C_{27}H_{29}N_3O$
Exact Mass: 411.23

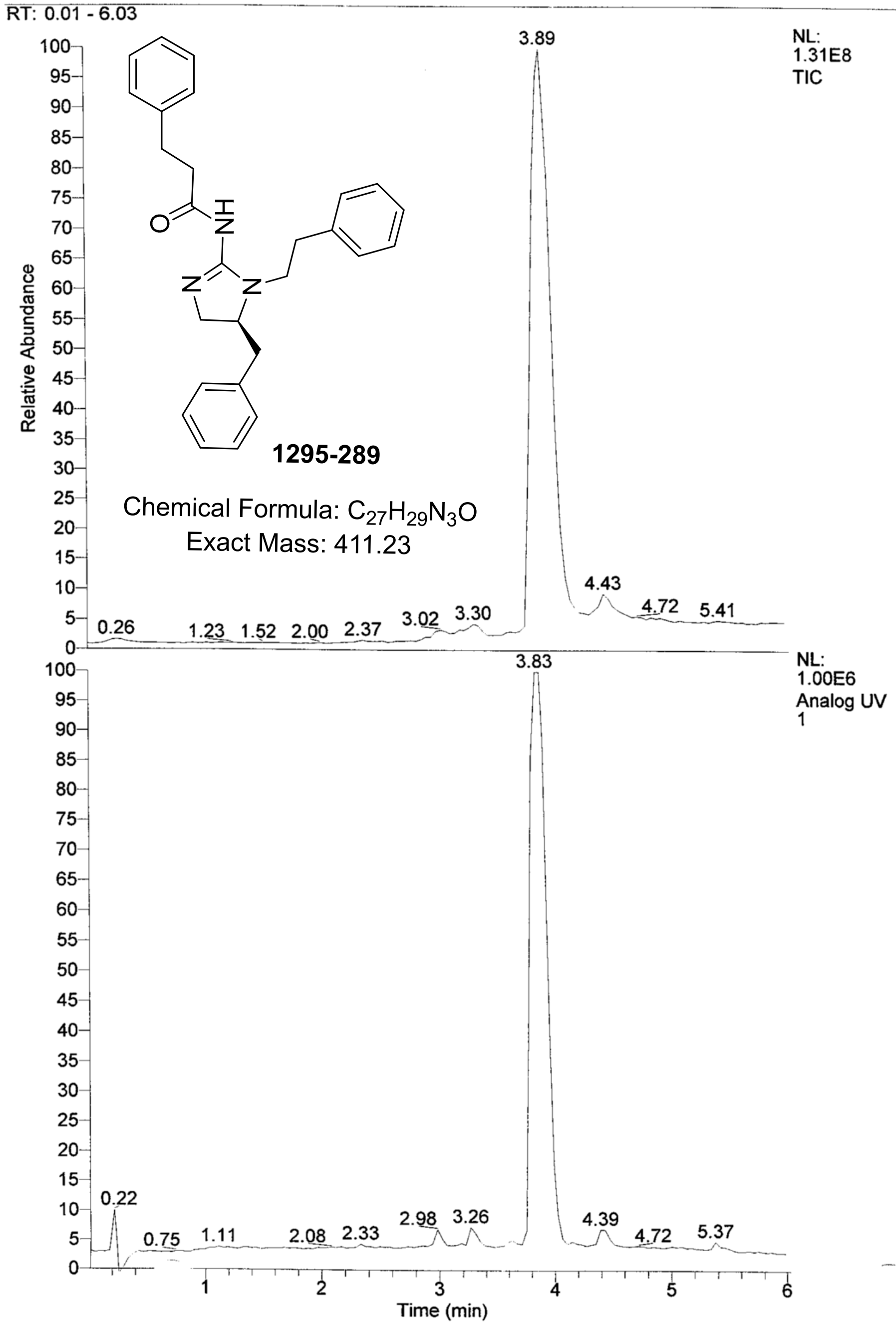

Experiment Method:
C:\LCQ\Methods\ESI\Short-col\ESI5-95m8 Created: 11/11/97
Creator: LCQ Last modified: 3/3/01 by
Administrator
Summary: 5-95m8
MS Run Time (min): 6.00

Autosampler Settings:

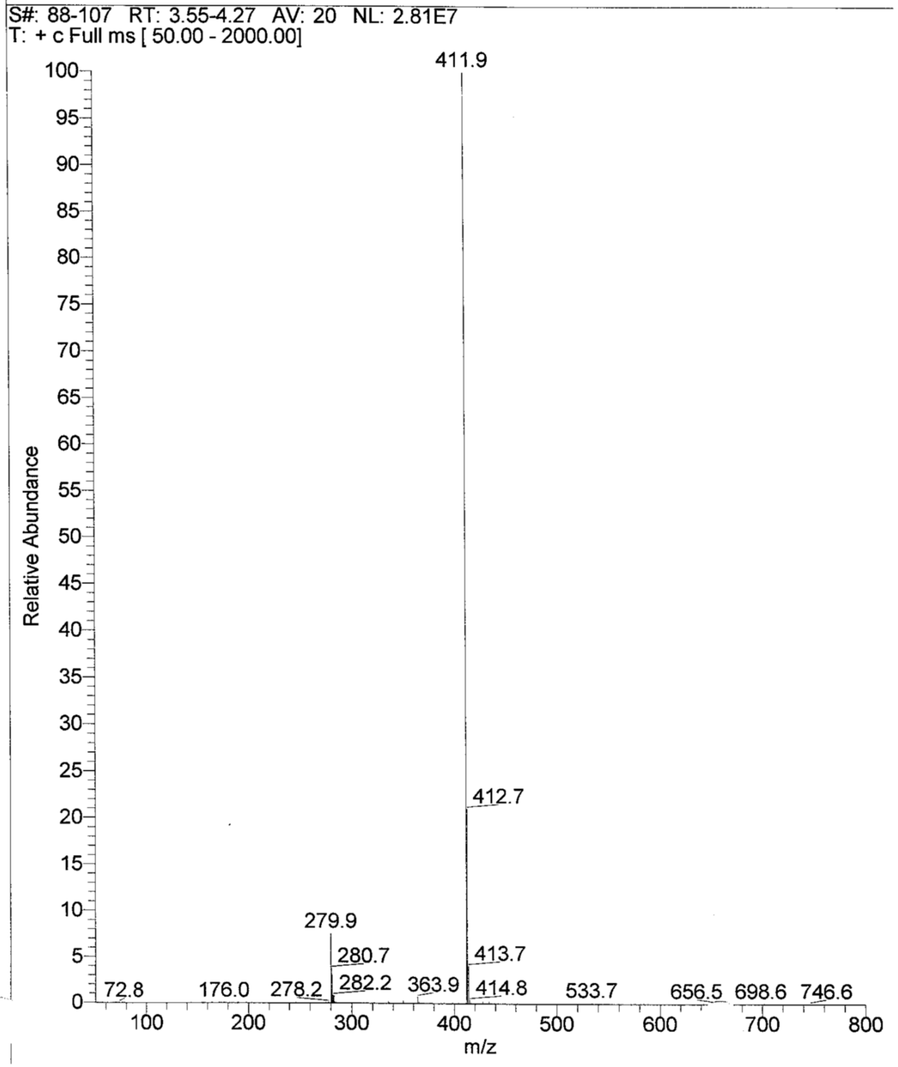

1295-298

Chemical Formula: $C_{23}H_{29}N_3O$

Exact Mass: 363.23

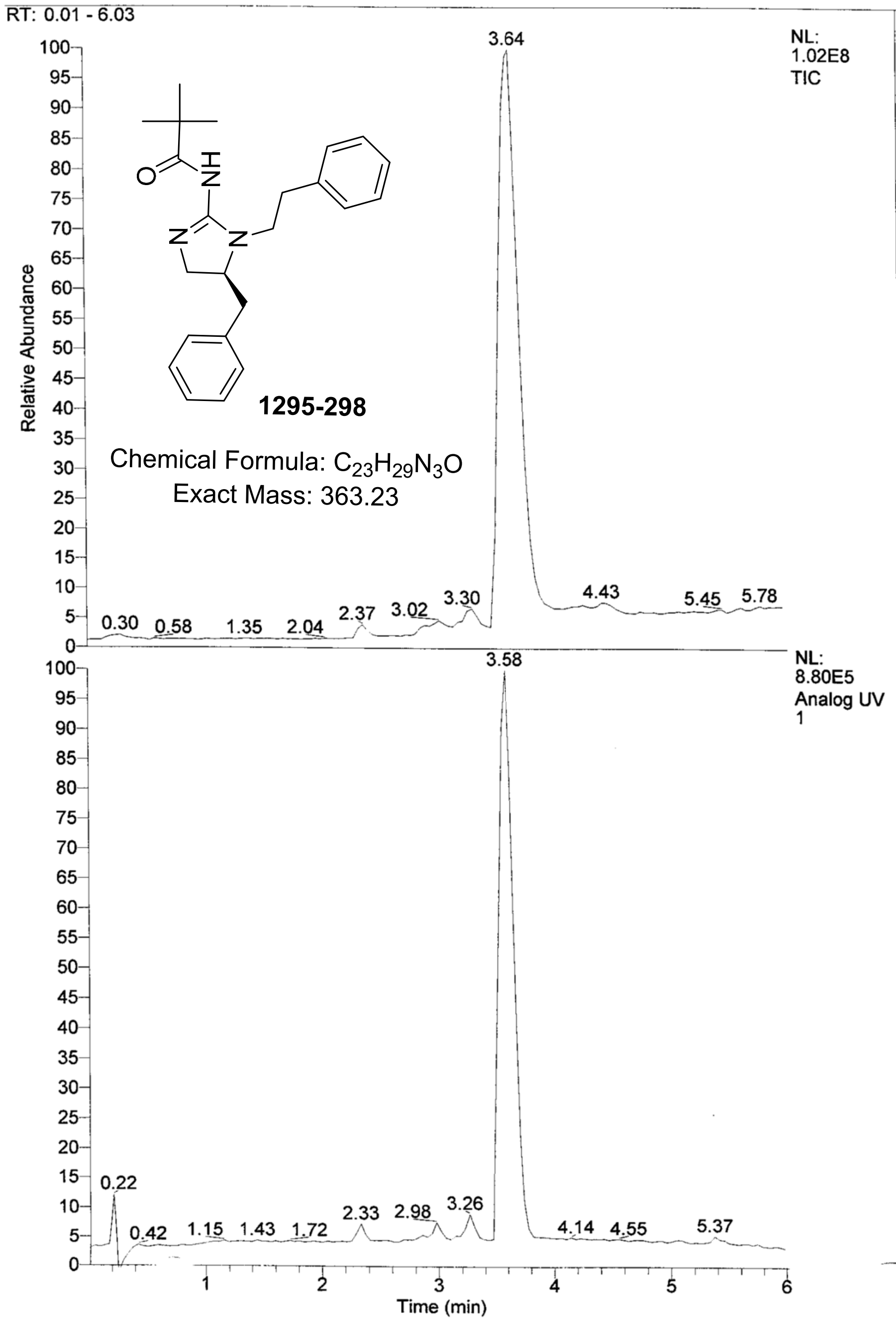


```
Experiment Method:
C:\LCQ\Methods\ESI\Short-col\ESI5-95m8  Created: 11/11/97
Creator: LCQ                          Last modified: 3/3/01 by
Administrator
Summary: 5-95m8
MS Run Time (min): 6.00

Autosampler Settings:
```

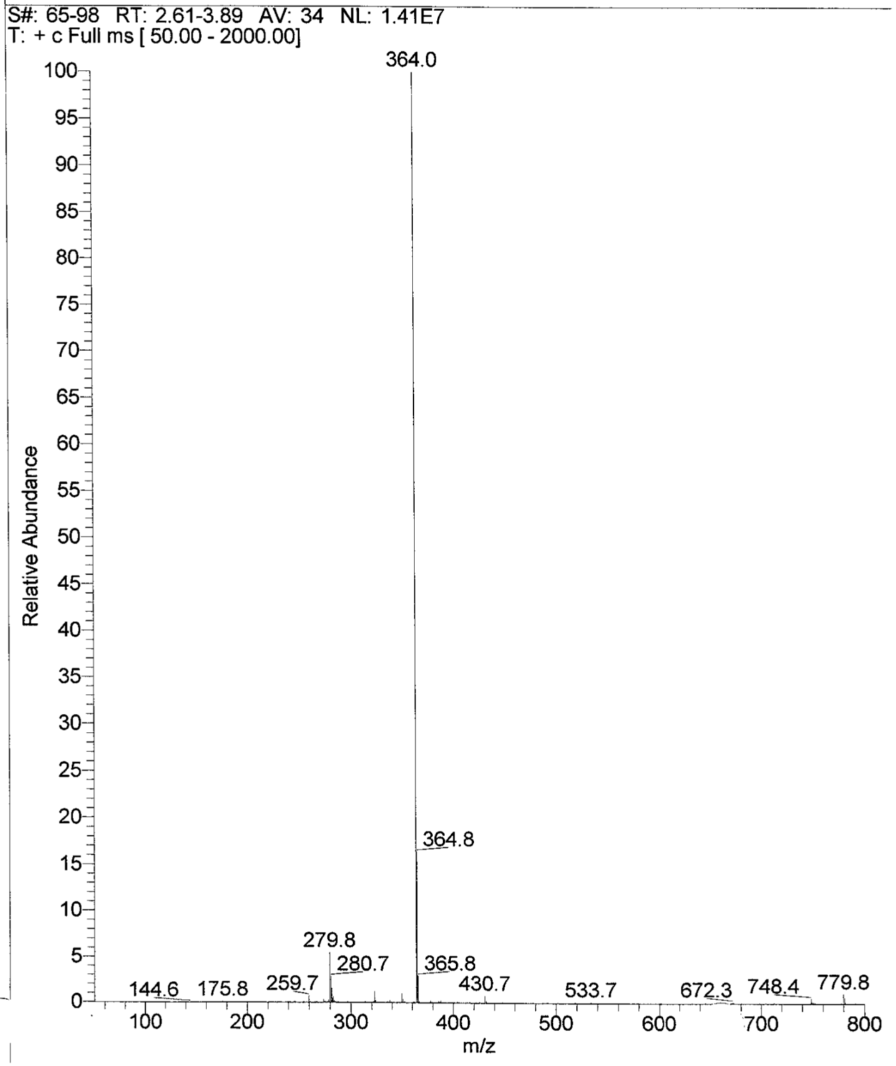

RT: 0.01 - 6.01

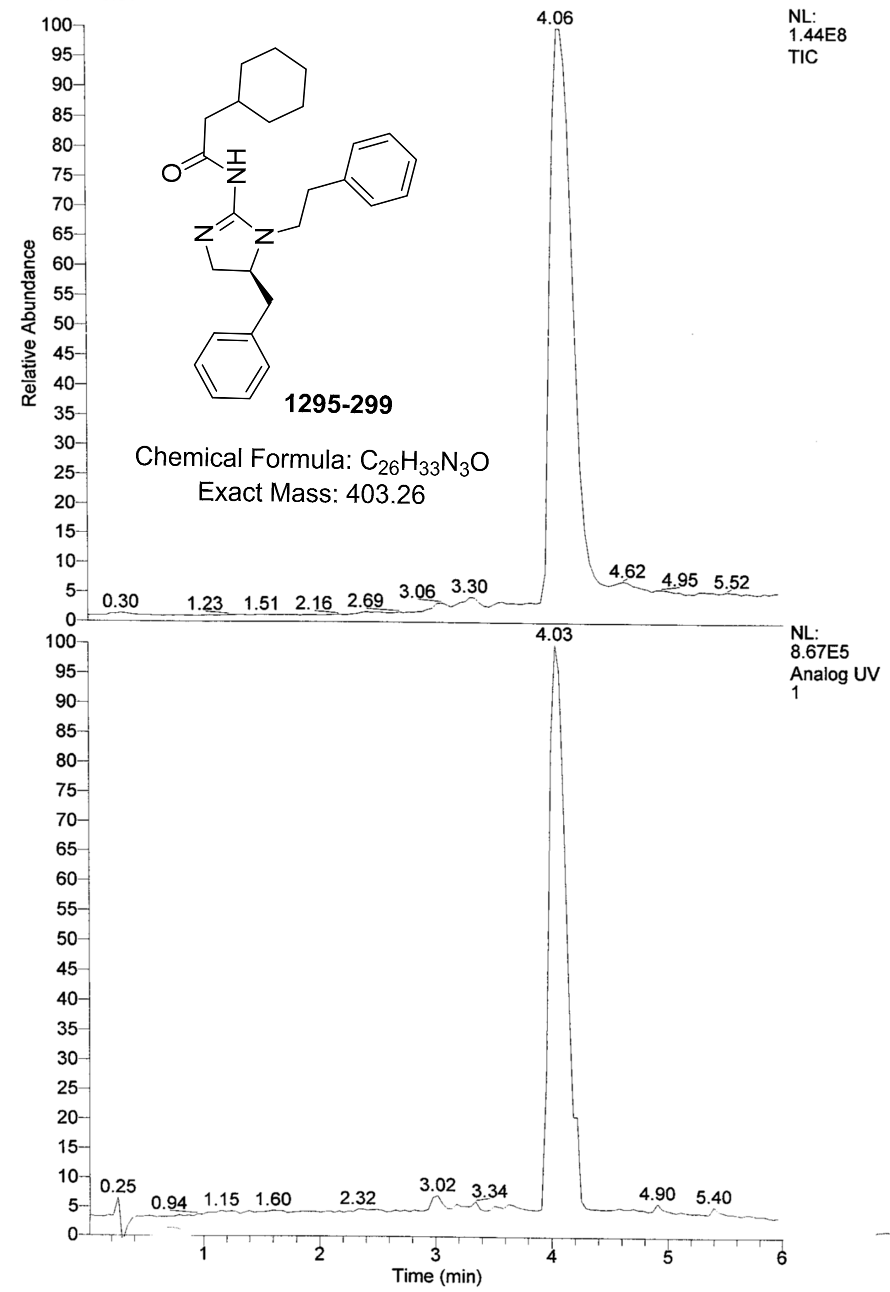

1295-299

Chemical Formula: $C_{26}H_{33}N_3O$

Exact Mass: 403.26

Experiment Method:
C:\LCQ\Methods\ESI\Short-col\ESI5-95m8 Created: 11/11/97
Creator: LCQ Last modified: 3/3/01 by
Administrator
Summary: 5-95m8
MS Run Time (min): 6.00

Autosampler Settings:

S#: 92-110 RT: 3.71-4.37 AV: 19 NL: 3.68E7
T: + c Full ms [50.00 - 2000.00]

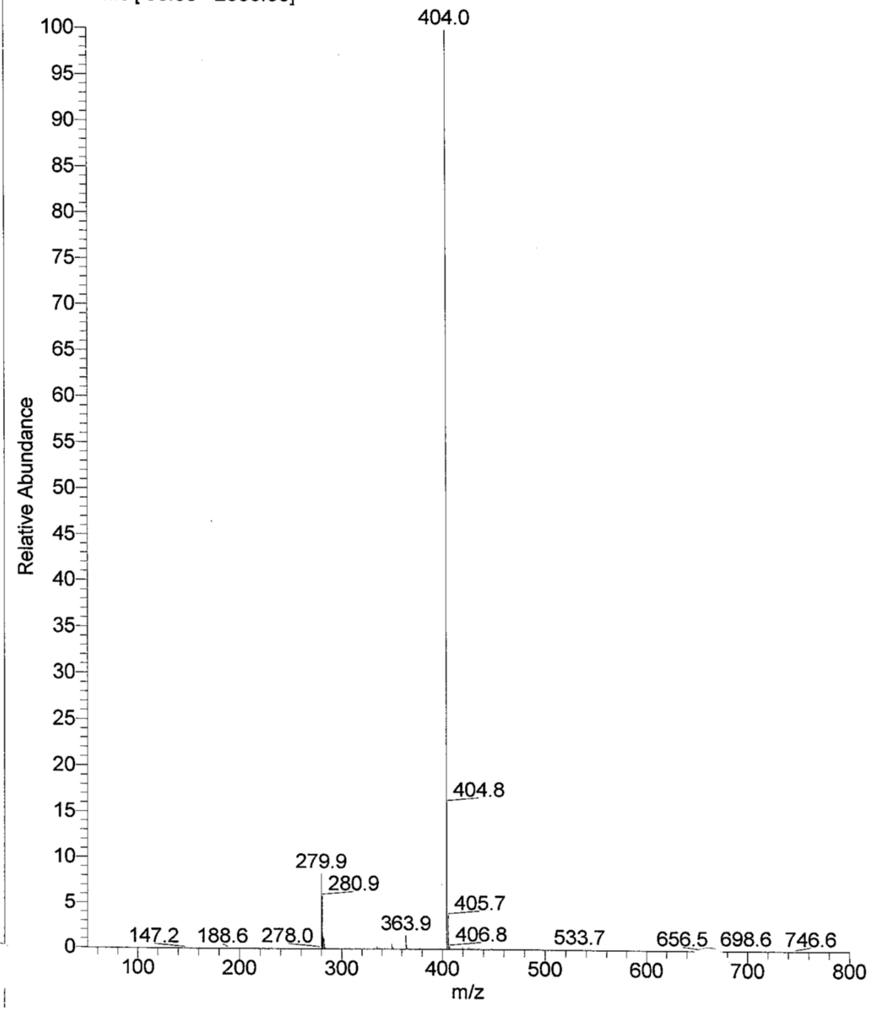

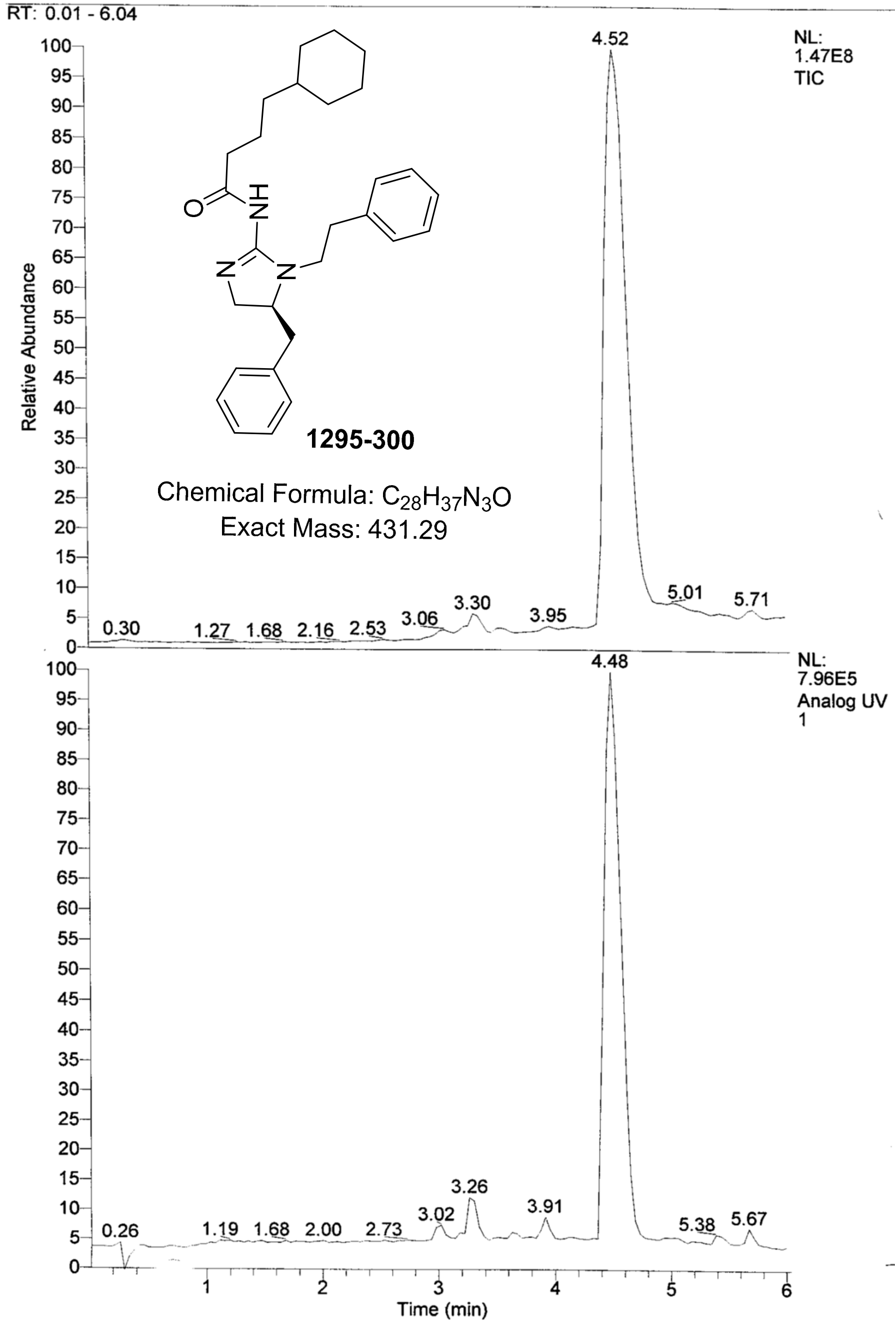

Experiment Method:
C:\LCQ\Methods\ESI\Short-col\ESI5-95m8 Created: 11/11/97
Creator: LCQ Last modified: 3/3/01 by
Administrator
Summary: 5-95m8
MS Run Time (min): 6.00

Autosampler Settings:

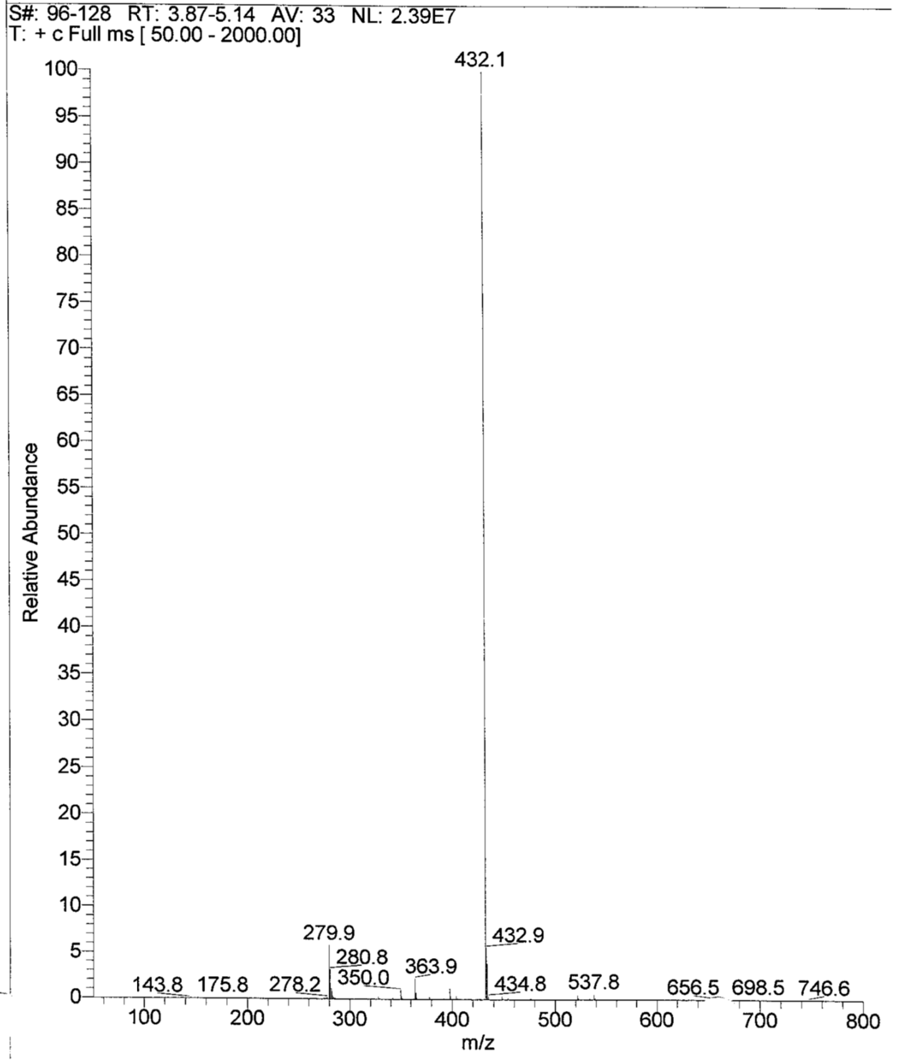

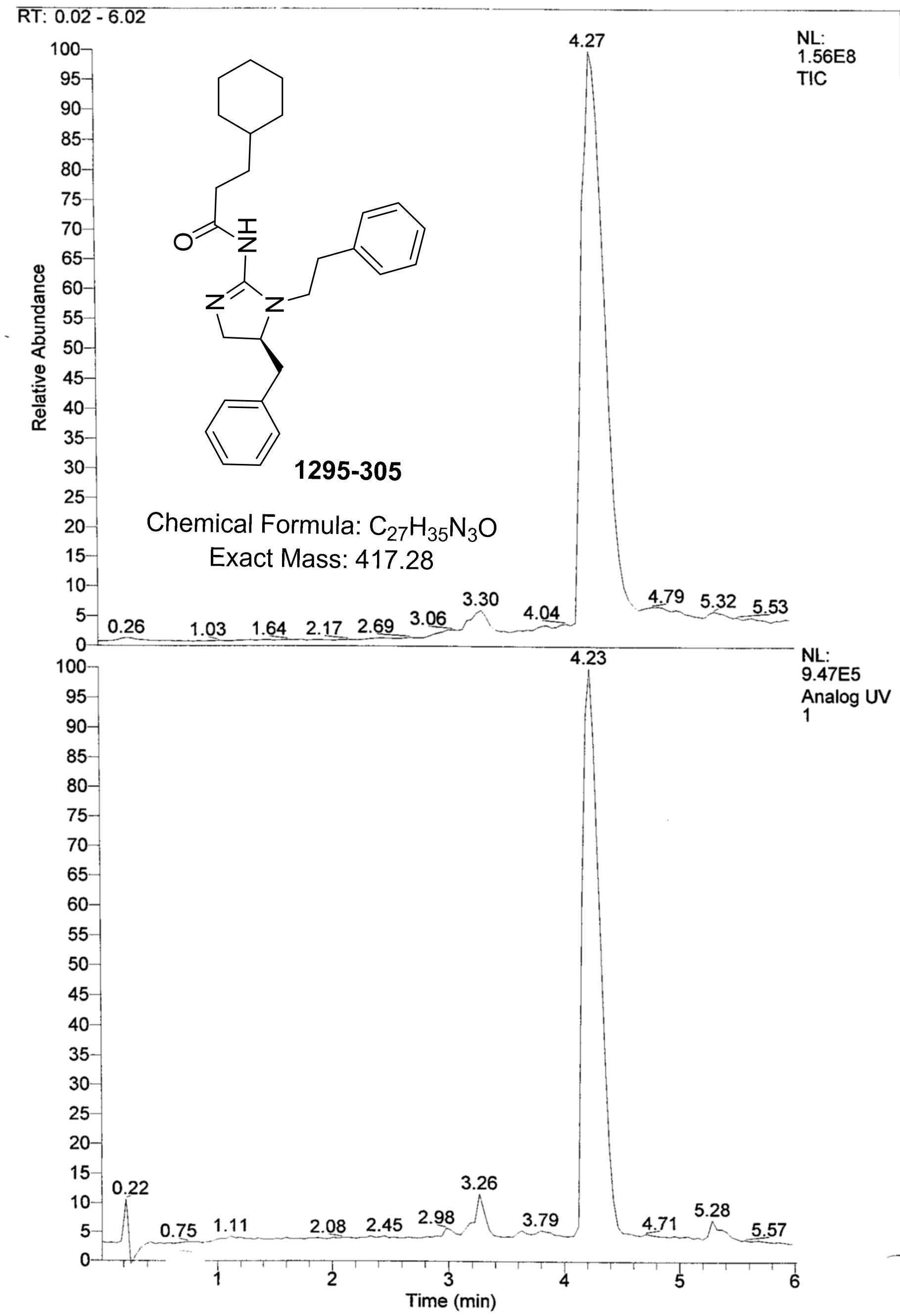

Experiment Method:
C:\LCQ\Methods\ESI\Short-col\ESI5-95m8 Created: 11/11/97
Creator: LCQ Last modified: 3/3/01 by
Administrator
Summary: 5-95m8
MS Run Time (min): 6.00

Autosampler Settings:

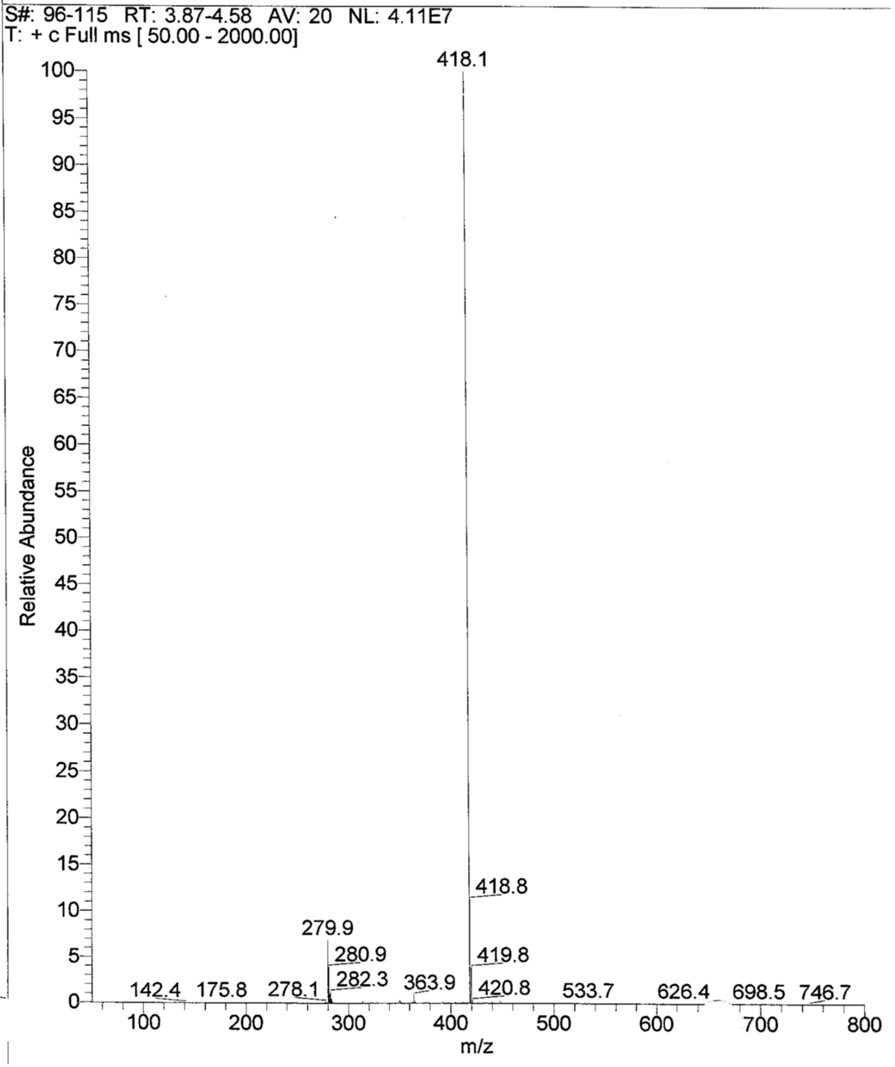

1295-306

Chemical Formula: $C_{26}H_{33}N_3O$

Exact Mass: 403.26

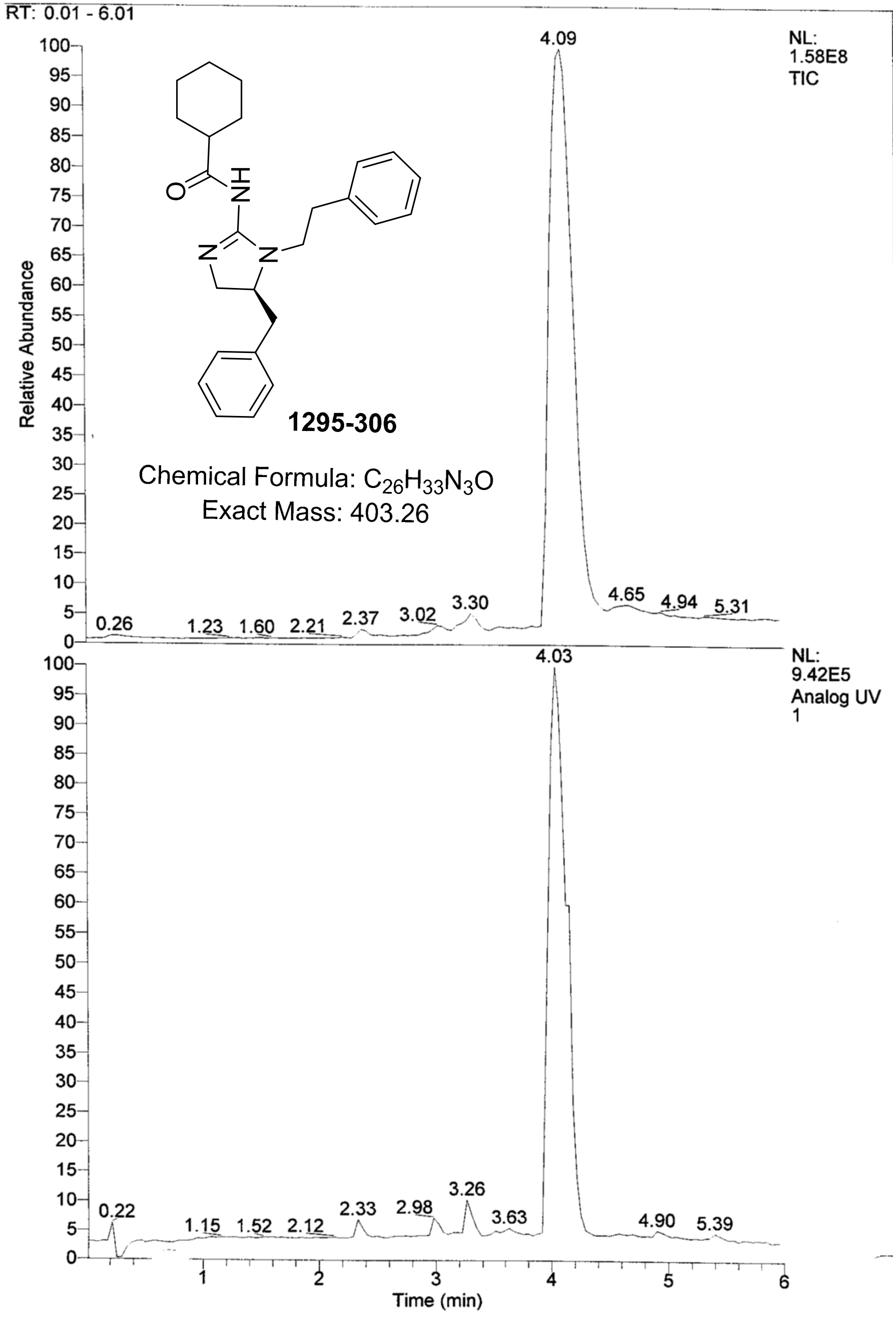

Experiment Method:
C:\LCQ\Methods\ESI\Short-col\ESI5-95m8 Created: 11/11/97
Creator: LCQ Last modified: 3/3/01 by
Administrator
Summary: 5-95m8
MS Run Time (min): 6.00

Autosampler Settings:

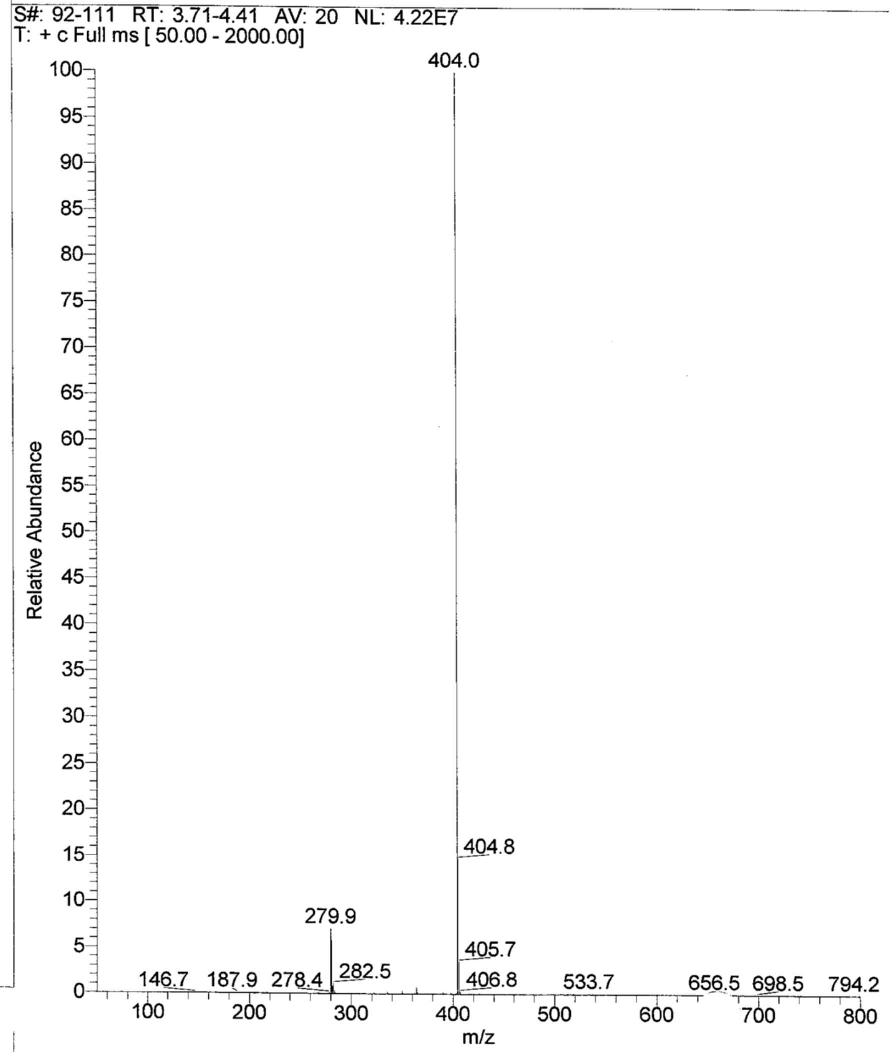

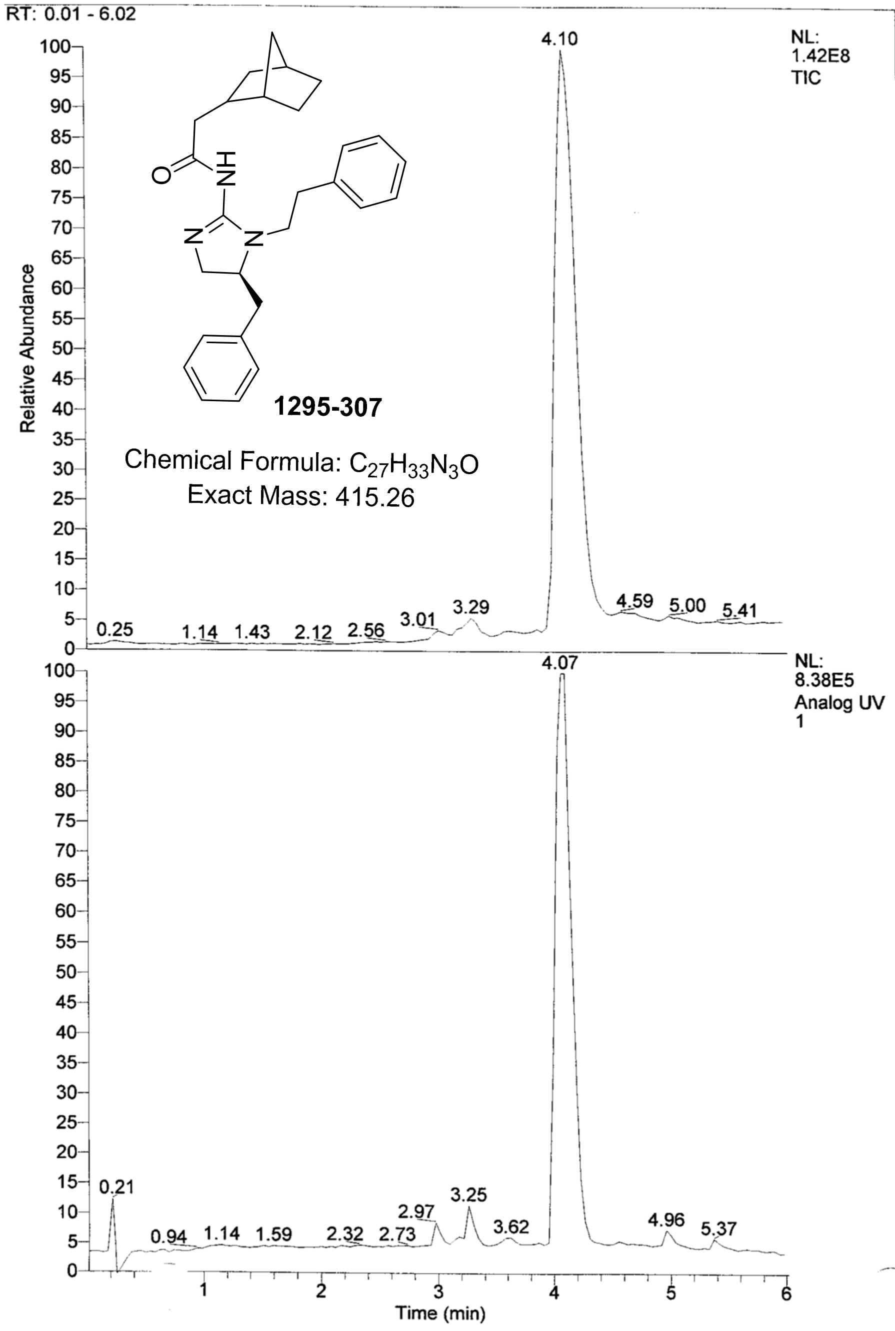

Experiment Method:
C:\LCQ\Methods\ESI\Short-col\ESI5-95m8 Created: 11/11/97
Creator: LCQ Last modified: 3/3/01 by
Administrator
Summary: 5-95m8
MS Run Time (min): 6.00

Autosampler Settings:

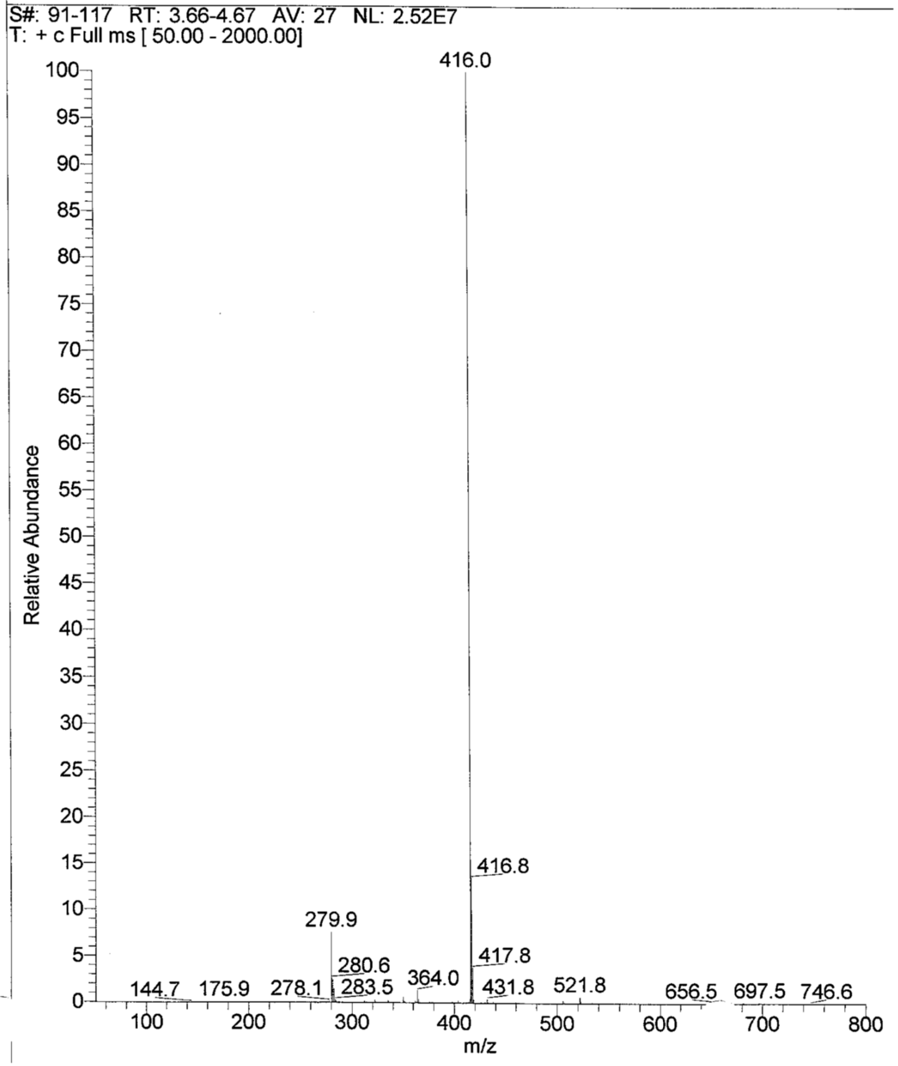

2520-31

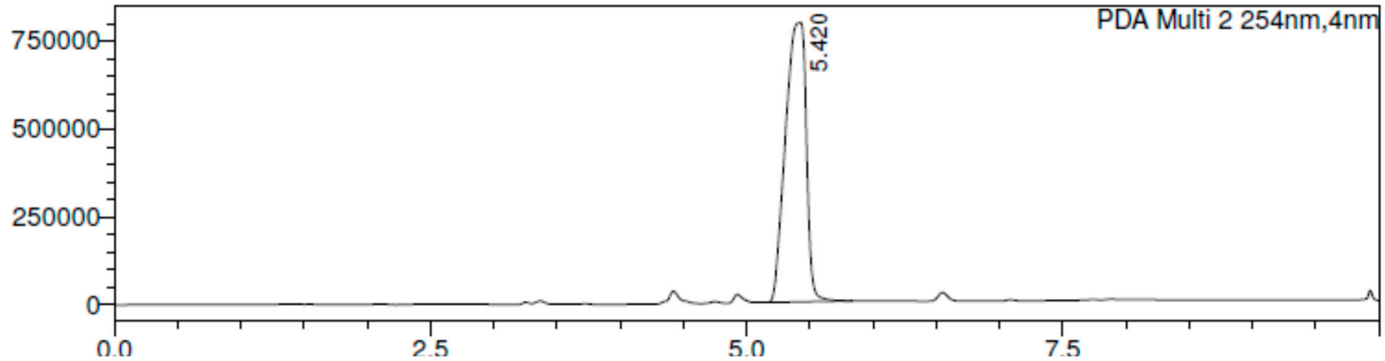

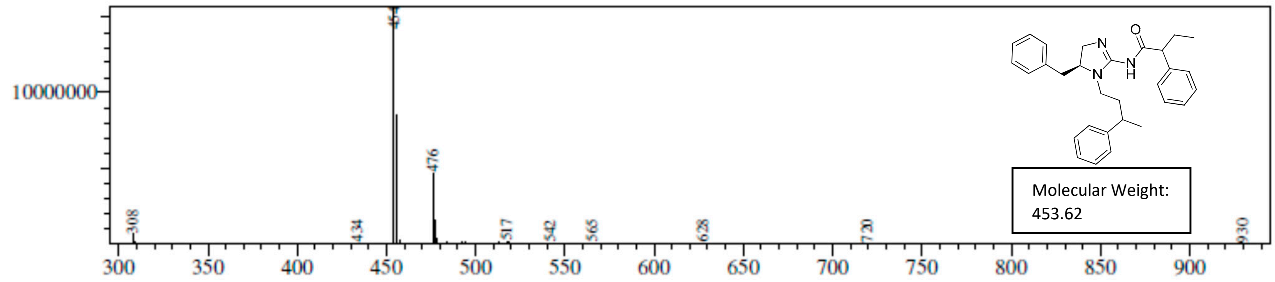

2520-39

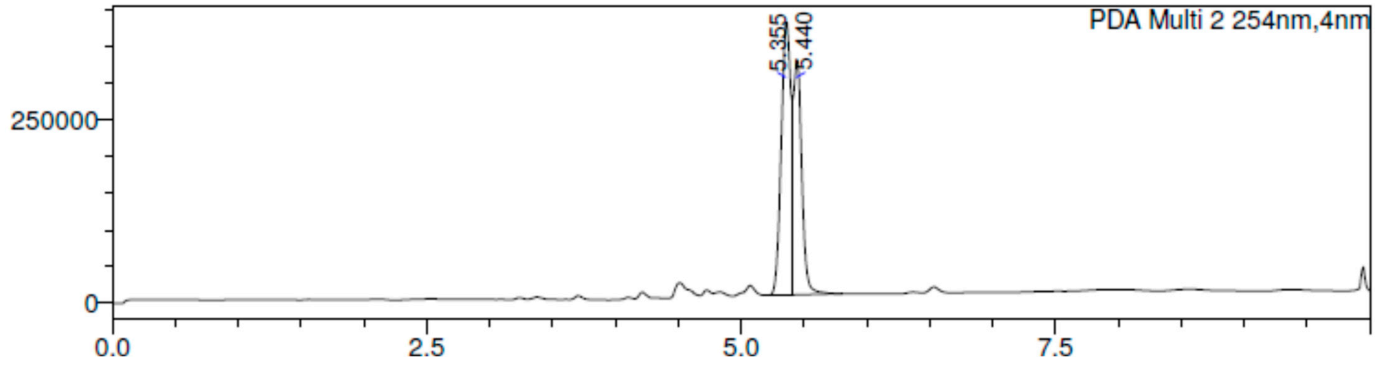

Diastereomeric mixture.

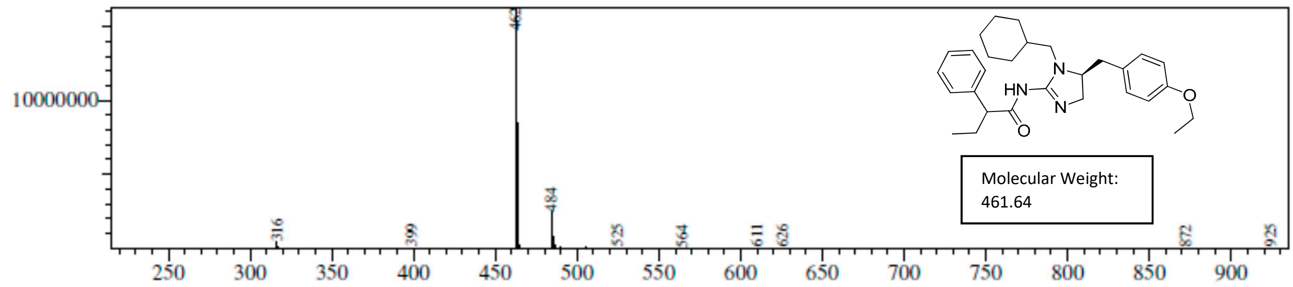

2520-47

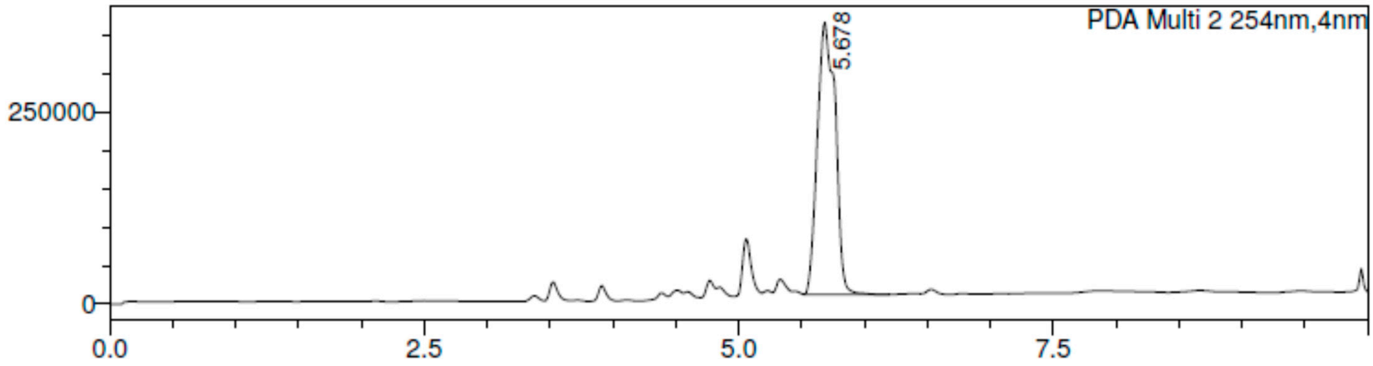
PDA Multi 2 254nm,4nm
5.678
250000
0
0.0
2.5
5.0
7.5

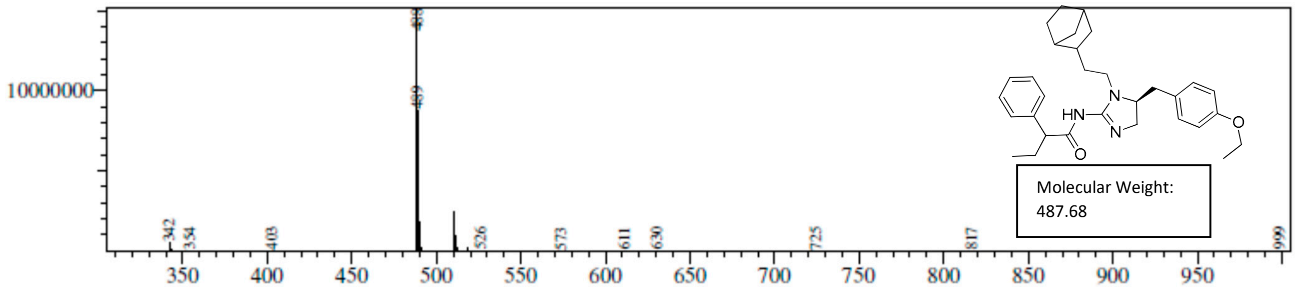
10000000
342
354
403
489
526
573
611
630
725
817
999
350
400
450
500
550
600
650
700
750
800
850
900
950
HN
N
N
O
O
Molecular Weight:
487.68
